# Deep transfer learning and attention based P2.5 forecasting in Delhi using a decade of winter season data

**DOI:** 10.1038/s41598-025-16664-4

**Published:** 2025-08-28

**Authors:** S. Lakshmi, A. Krishnamoorthy

**Affiliations:** School of Computer Science Engineering, Vellore Institute of Technology, Vellore, 632014 India

**Keywords:** Air quality prediction, *PM*_2.5_ prediction, Deep learning, Multi-head attention, Transfer learning, Environmental sciences, Environmental social sciences, Environmental economics, Computational science, Computer science

## Abstract

This investigation focuses on the phenomenon of air pollution in the metropolitan area of Delhi, with a particular emphasis on the stubble-burning season, during which concentrations of *PM*_2.5_ reach their peak, presenting significant health hazards. Utilizing a comprehensive dataset spanning a decade (2012–2022), this study analyzes the influence of meteorological conditions, urban emissions, and seasonal biomass combustion. It amalgamates historical *PM*_2.5_ concentration data, relevant meteorological variables, and FIRECOUNT data to capture the temporal and pollution dynamics. Feature selection based on CorrXGBoost was utilized to find and keep the most significant predictors, hence decreasing model complexity while maintaining predictive efficacy. The proposed hybrid TL-LSTM-MHA Long Short-Term Memory (LSTM) model, augmented with Multi-Head Attention, is employed, harnessing transfer learning techniques to facilitate enhanced computational efficiency and generalization capabilities. The model demonstrated good performance (MAE = 4.38, RMSE = 5.80, R^2^ = 0.9972) and was extensively verified using tenfold cross-validation to ensure robustness towards overfitting and non-stationary effects. Statistical significance tests, particularly the Wilcoxon signed-rank test, were used to confirm the performance disparities among model variations, therefore substantiating the roles of essential architectural elements. Attention weight visualization and head-wise interpretability studies demonstrated unique patterns in feature significance across heads. The model’s efficacy was also assessed against traditional and contemporary state-of-the-art methods tested on similar *PM*_2.5_ forecasting tasks, demonstrating its enhanced accuracy. This research provides predictive insights pertinent to regulatory decision-making about seasonal air quality management encountered in Delhi. The scalability of the proposed framework is demonstrated by comparing it to conventional and transfer learning-based models.

**Supplementary Information:**

The online version contains supplementary material available at 10.1038/s41598-025-16664-4.

## Introduction

As academics emphasize, air pollution is a critical worldwide challenge with far-reaching effects on welfare, the environment, and economic growth. Cities such as Delhi in India have very elevated pollution levels, underscoring the severity of these issues^1,2^. The Air Quality Index (AQI) is determined by measuring several pollutants, including particulate matter (*PM*_2.5_ and PM10) and Ozone (O3), Nitrogen Dioxide (NO2), Sulphur Dioxide (SO2), and Carbon Monoxide (CO) emissions^3,4^ Absorption of pollutants such as *PM*_2.5_, PM10, NO2, SO2, CO, and O3 is correlated with respiratory and cardiovascular disorders, premature mortality, and environmental consequences, including global warming and the release of greenhouse gases. Biomass combustion is a sustainable source of airborne particulate matter (PM) and chemical gases, which profoundly influence both local and global climates. It also presents significant health hazards to people. This type of combustion encompasses various activities, including wildfires and post-harvest agricultural burning, commonly referred to as crop residue burning (CRB) or “stubble” burning^5^. Stubble burning in North India, a practice that has been conducted for more than twenty years, involves farmers in Punjab, Haryana, Uttar Pradesh, and adjacent states incinerating agricultural remnants after harvest to expedite soil preparation for subsequent planting^3^. This practice, particularly from September to December, deteriorates air quality as winds transport smoke and pollutants, such as *PM*_2.5_, PM_10_, NO_2_, SO_2_, CO, and O_3_, to the National Capital Territory and other areas, resulting in hazardous smog^6^. Air pollution in Delhi intensifies throughout the winter, attributed to the Diwali celebrations and the incineration of agricultural waste, which is exacerbated by reduced temperatures and heightened heating requirements. Pollution is less during the monsoon; however, it remains considerable. Interest in the subject intensifies throughout winter, as seen by media coverage, public engagement, and political discourse^7^. Over the last decade, numerous studies have examined the temporal dynamics of air quality, emphasizing the influence of meteorological parameters, including wind speed (WS), relative humidity (RH), and wind direction (WD), on pollutant levels. The stubble-burning season presents distinct challenges due to the complex interplay of meteorological variables, agricultural practices, and urban pollutants. This requires the creation of sophisticated forecasting models, particularly designed for this timeframe. Nevertheless, most current research lacks models particularly designed for the stubble-burning season and frequently encounters issues with data scarcity and seasonality, resulting in overfitting in deep learning applications. Furthermore, these models often fail to account for the sudden, significant surges in *PM*_2.5_ concentrations that result from biomass burning in adjacent areas. They often rely on smooth seasonal patterns, overlook real-time fire activity, and fail to account for the intricate climatic dynamics—such as temperature inversions and low wind speeds—common during the post-monsoon period. Moreover, many fail to utilize the capabilities of transfer learning or sophisticated attention processes to elucidate intricate spatiotemporal correlations in air quality data.

To address these challenges, this research aims to develop a seasonal *PM*_2.5_ forecasting model for the stubble-burning period (September–December) using a decade of historical air quality and meteorological data (2012–2022). A Transfer Learning-based LSTM with Multi-Head Attention (TL-LSTM-MHA) is introduced, pre-trained on historical (source) data and subsequently fine-tuned on recent (target) data to improve generalization. A hybrid feature selection approach (CorrXGBoost), integrating Pearson correlation with Gradient Boosting significance, determines the most pertinent predictors. This spatiotemporal paradigm allows precise forecasting of *PM*_2.5_ surges during stubble-burning events. This study provides policymakers with empirically derived forecasts, promoting innovation and sustainable practices that benefit both agricultural farmers and urban residents in the National Capital Region. The work efficiently addresses data shortages and improves model robustness in a highly seasonal situation through the application of transfer learning.

The explicit objectives consist of:

1. *Seasonal air quality prediction* Establish a dedicated system for precisely forecasting *PM*_2.5_ levels through the pivotal stubble-burning season, marked by elevated pollutant levels and health hazards.
2. *Temporal dynamics and feature enrichment* Integrated lagged *PM*_2.5_ readings, rolling statistics, as well as seasonal climate data, including wind speed (WS), relative humidity (RH), and wind direction (WD), to elucidate temporal and meteorological effects. Furthermore, using FIRECOUNT data to assess the regional effects of agricultural residue combustion.
3. *Long-term trends analysis* Employ a decade (2012–2022) of seasonal data to simulate long-term trends and variability in *PM*_2.5_ concentration affected by meteorological conditions, agricultural residue combustion, and urban emissions.
4. *Advanced deep learning model* Propose a hybrid LSTM and Multi-Head Attention model to capture pattern sequences. The model emphasizes significant time steps in the data, enhancing the accuracy of predictions.
5. *Transfer learning for efficiency* Employs a two-phase strategy: initially, the model is pre-trained on historical source data prior to 2021 to identify temporal and pollutant-related patterns, followed by fine-tuning on the target data from 2021 onward. This method improves generalization, expedites training, and decreases computing expenses by leveraging acquired temporal representations.
6. *Feature selection for model simplification and interpretability* A CorrXGBoost-Rank-based feature selection technique integrates correlation analysis with XGBoost significance scoring to ascertain the most pertinent predictors of *PM*_2.5_ concentrations. The chosen characteristics are subsequently utilized as inputs to the TL-LSTM-MHA architecture, which enhances model interpretability, reduces input dimensionality, and promotes learning efficiency during the stubble-burning season.
7. *Quantitative evaluation* Assess the model’s performance using rigorous measures such as MAE, RMSE, and R2 to guarantee correctness and dependability.
8. *Policy implications* Deliver accurate seasonal forecasts to policymakers and ecological authorities to enable prompt actions and alleviate the health and air quality repercussions of stubble burning on Delhi’s air quality.
9. *Comparative performance and scalability* Assess the model’s scalability and robustness by evaluating its performance relative to traditional and baseline models trained on the identical dataset. Furthermore, show its applicability to structurally analogous seasonal scenarios utilizing pre-trained weights.

The study is arranged systematically as follows: Sections “Introduction”, “Related work”, “Materials and methods”, “Experimental details”, “Results”."Discussion and Future Work","Conclusion"

## Related work

As contaminants are dynamic, uncertain, and extremely unpredictable, predicting air quality is difficult. Conventional deterministic approaches are not flexible enough to adjust to changing circumstances and are predicated on assumptions^8^. Although statistical methods are more flexible, their ability to handle the non-linear character of real-world data is limited since they frequently make linear assumptions^9^.Significant scholarly inquiry undertaken by Ameer et al. investigated the efficacy of four distinct regression methodologies: Decision Tree, Gradient Boosting, Multilayer Perceptron, and Artificial Neural Network, which are employed to predict air quality indices^10,11^.

Big data analysis has been greatly enhanced by deep learning (DL), a complex machine learning subfield in several fields, including biological informatics, speech recognition, visual analytics, and remote sensing. By learning in-depth via several phases, DL excels at non-linear resolving issues, and its effectiveness becomes better as the dataset size grows. DL approaches have been effectively used to solve a variety of issues, such as voice analysis, motion modeling, picture classification, object recognition, weather forecasting, and natural language processing^12^. Given the volume of air pollution data, it makes sense. It works well to use DL models in conjunction with cutting-edge AI techniques to accurately depict and forecast air quality depending on weather and other variables^13^.

Hours to weeks are only a few of the short and long-term effects that air pollution may have on the ecosystem and human health. Consequently, while forecasting air quality, temporal delays must be considered. Nevertheless, a lot of Artificial Neural Networks (ANN)^14^-based techniques have trouble establishing long-term relationships or successfully addressing the temporal delays of air pollution. Recurrent neural (RNNs)^15^, long short-term memory (LSTM) models^16–19^. LSTM incorporated into fully connected neural networks (LSTM-FC)^20^. Combination models, such as K-nearest neighbor with LSTM (KNN-LSTM), are sophisticated methods for deep learning that some researchers have used to model time series data to get around these restrictions.

Despite its severe pollutant spikes and related health hazards, air quality forecast techniques often lack real-time, season-specific modeling designed for high-pollution times, including stubble-burning season. Additionally, health hazards are rarely included in existing frameworks for thorough seasonal forecasts. In addition, air quality studies usually just look at whether or temporal aspects, ignoring an integrated strategy that uses metrics like FIRECOUNT for regional pollution evaluation, despite the progress achieved in the domain of feature selection, the prevailing methodologies continue to exhibit significant limitations.

Su et al.^21^ and Farhani^22^ concentrated their efforts on predicting fire risk, however, they failed to incorporate integrated with delayed *PM*_2.5_ measurements, rolling statistics, and seasonal climate data. Both the effect of climate change on seasonal *PM*_2.5_ fluctuation and the cumulative impact of burning crop residue and urban pollutants over long periods are still poorly understood. In collecting wider contextual linkages, the self-attention mechanism greatly improves series processing and gets beyond the drawbacks of conventional techniques that rely on brief windows for aggregating past material^23^. Its capacity to extract important information from input matrices is further improved by regularization terms. By facilitating the concurrent aggregation of many linear transformations, the multi-head self-attention system expands on these advantages and successfully captures complex trends and connections.

Utilizing this technique, air quality forecasting fills in the gaps in the computation of intricate temporal relationships and interactions that are frequently missed by conventional methods^24^. Hybrid frameworks such as LSTM combined with Multi-Head Attention for selecting important steps in data are still not completely utilized by sophisticated deep learning models. An important development in AI and deep learning is transfer learning (TL)^25^, which improves learning and forecasting effectiveness by enabling a pre-built model to transfer information from a source job to a similar target task^26^. This method enhances model accuracy and generalization and works especially well in situations with little training data. TL is helpful in a variety of fields, such as building usage, neurophysiological research, and environmental research, since it reuses existing information, unlike classical machine learning, which creates every prediction from the start. By being pre-trained modestly, it has demonstrated particular use in data-poor settings for air pollution forecasting, allowing for increased forecast accuracy. Prasanthrajan et al.^27^ illustrated that tree species exhibited considerable physiological diversity between polluted and unaffected areas within the same city, underscoring local and temporal disparities in environmental stress. This substantiates the justification for implementing transfer learning within a singular domain, wherein temporal variations can engender disparate learning contexts despite common geography.

The base manuscript investigates the application of Transfer Learning-oriented Hybrid Deep Learning methodologies for the prediction of *PM*_2.5_ concentrations, effectively addressing the challenge of data scarcity through the utilization of temporal attention mechanisms. This approach demonstrates superior performance compared to conventional models, achieving a reduction in RMSE of up to 38% on datasets from Beijing and Hengshui^28^.

This research expands upon this work by incorporating Long Short-Term Memory (LSTM) networks with Multi-Head Attention (MHA), CorrXGBoost based feature selection, Seasonal climate indicators and FIRECOUNT-derived spatial cues to enhance feature representation and forecast accuracy, to improve both feature representation and forecast precision. Furthermore, despite the possibility of shorter training times and increased flexibility, transfer learning has not yet been widely used in air quality prediction due to difficulties in balancing adaptation and efficiency. Furthermore, a TL-LSTM-MHA model is implemented to augment forecasting precision, incorporating seasonal climate data, FIRECOUNT metrics, and the effects of pollutant accumulation. This theoretical framework significantly advances the forecasting of long-term air quality. It enhances predictive accuracy in sparse data regions through the optimization of feature selection, the application of Multi-Head Attention (MHA) for the identification of patterns, and the amalgamation of deep learning techniques with transfer learning methodologies.

## Materials and methods

The materials and methods aspect of this research encompasses the following components: Study Area, Data Exploration and Preprocessing, and methods, which delineate the techniques, including the principles of Transfer Learning, the Multi-Head Attention mechanism, and the combined TL-LSTM-MHA modelling framework. Furthermore, it provides the requisite foundational information crucial for comprehending the proposed paradigm.

### Study area

This research uses a dataset combining air pollution metrics, economic indicators, and field fire data to predict air pollution levels in New Delhi, focusing on the period from September to December between 2012 and 2021. Air pollution data was gathered from five stationary monitoring stations—Anand Vihar, ITO, Mandir Marg, Shadipur, and R.K. Puram, which include 24-h averages of *PM*_2.5_, PM10, CO, NO2, and SO2^29^. The chosen stations ensure representative spatial coverage across this distribution. This distribution ensures comprehensive coverage of industrial, residential, and high-traffic sectors, facilitating more thorough modeling. Meteorological data comprises RH, WS, WD, SR, BP, and AT, with WS exhibiting a notable negative association with *PM*_2.5_. Data on field fires were sourced from NASA’s VIIRS 375 m Active Fire Data, concentrating on Punjab and Haryana, with FIRECOUNT reflecting daily fire occurrences during the stubble-burning season^30^. While FIRECOUNT does not quantify fire intensity, it consistently indicates seasonal patterns. Economic statistics, GSDP, and HDI values for New Delhi were incorporated as yearly constants. All data were consolidated into five station-specific files for regression and forecasting. Table 1. Shows the explanation of the study’s attribute feature.

**Table 1:** Explanation of the study’s attribute feature.

No of feature	Feature	Description of dataset	Datatype
1	DATE	Date of observation	object
2	*PM*_2.5_	Particular matter diameter of 5	float64
3	PM10	Particular matter diameter of 10	float64
4	NO2	Nitrogen dioxide	float64
5	CO	Carbon monoxide	float64
6	SO2	Sulfur dioxide	float64
7	FIRECOUNT	No of fire incidents	int64
8	WD	Wind direction	float64
9	WS	Wind seed	float64
10	RH	Relative humidity	float64
11	AT	Ambient temperature	float64
12	BP	Atmospheric pressure	float64
13	SR	Solar radiation	float64
14	CONST	Constant variable	int64
15	GSDP	Gross state domestic product	int64
16	GSVA	Gross state value added	float64
17	GSDP_CAP	Gross state domestic product per capita	int64
18	HDI	Human development index	float64
19	*PM*_2.5__lag_1	*PM*_2.5_ value 1 steps prior	float64
20	*PM*_2.5__lag_2	*PM*_2.5_ value 2 steps prior	float64
21	*PM*_2.5__lag_3	*PM*_2.5_ value 3 steps prior	float64
22	*PM*_2.5__rolling_mean	Rolling average of *PM*_2.5_ over a defined window	float64
23	*PM*_2.5__rolling_std	Rolling std of *PM*_2.5_ over a defined window	float64
24	Month	Month of year	int64
25	Day_of_week	Day of the week	int64

The data was obtained from Agarwal, Arti (2022). Data for: The Economic Cost of Air Pollution Due to Stubble Burning: Evidence from Delhi Version 1. Mendeley Data, October 3, 2022. Available at: 10.17632/yxzxvxtvpr.1. ^31^ This comprehensive dataset provides a strong foundation for studying the relationship between agricultural fires and guiding air quality policy. Table 1 explains the study’s attribute features.

### Data exploration and preprocessing

This section outlines the essential steps for refining the dataset to achieve effective modeling. It covers handling missing values, eliminating redundancies, analyzing the impacts of fire incidents, scaling with Temporal-Enhanced Feature Engineering (TEFE), testing and correcting stationarity, normalizing the data, and selecting features to improve model performance. The procedures for data cleaning, preprocessing, and feature transformation are elaborated in Supplementary File S1 (S1_Data_Preprocessing.ipynb).

#### Missing values handling

Addressing missing data is essential for preparing datasets for reliable analysis and modeling. This study began by analyzing the dataset to evaluate the extent of missing data across all 18 aspects, including both continuous and categorical variables. Many meteorological and pollution-related variables—such as PM10, *PM*_2.5_, CO, NO2, SO2, WD, RH, WS, AT, GSVA, and HDI—showed varying levels of missing data, ranging from 3% to over 25%. Linear interpolation was applied to continuous time-series variables (e.g., *PM*_2.5_, WS, RH, AT) to maintain temporal consistency in sequential data. For variables with little temporal dependence or moderate missingness (e.g., GSVA, HDI), mean imputation was used to minimize bias while preserving feature distribution. Categorical or directional variables, such as WD, were imputed using mode substitution to retain the most common value and preserve categorical integrity. Variables with substantial missing data—such as BP (84%), SR (86%), and CONST (with 243 missing entries)—were excluded due to insufficient coverage and their potential to harm model performance. After preprocessing, the dataset with imputed values was revalidated to confirm the absence of missing data across all remaining variables. This systematic approach ensured data integrity across both continuous and categorical variables, enabling the reliable use of data for rigorous temporal modeling and predictive analysis.

#### Removal of redundant features

**Fig. 1:**
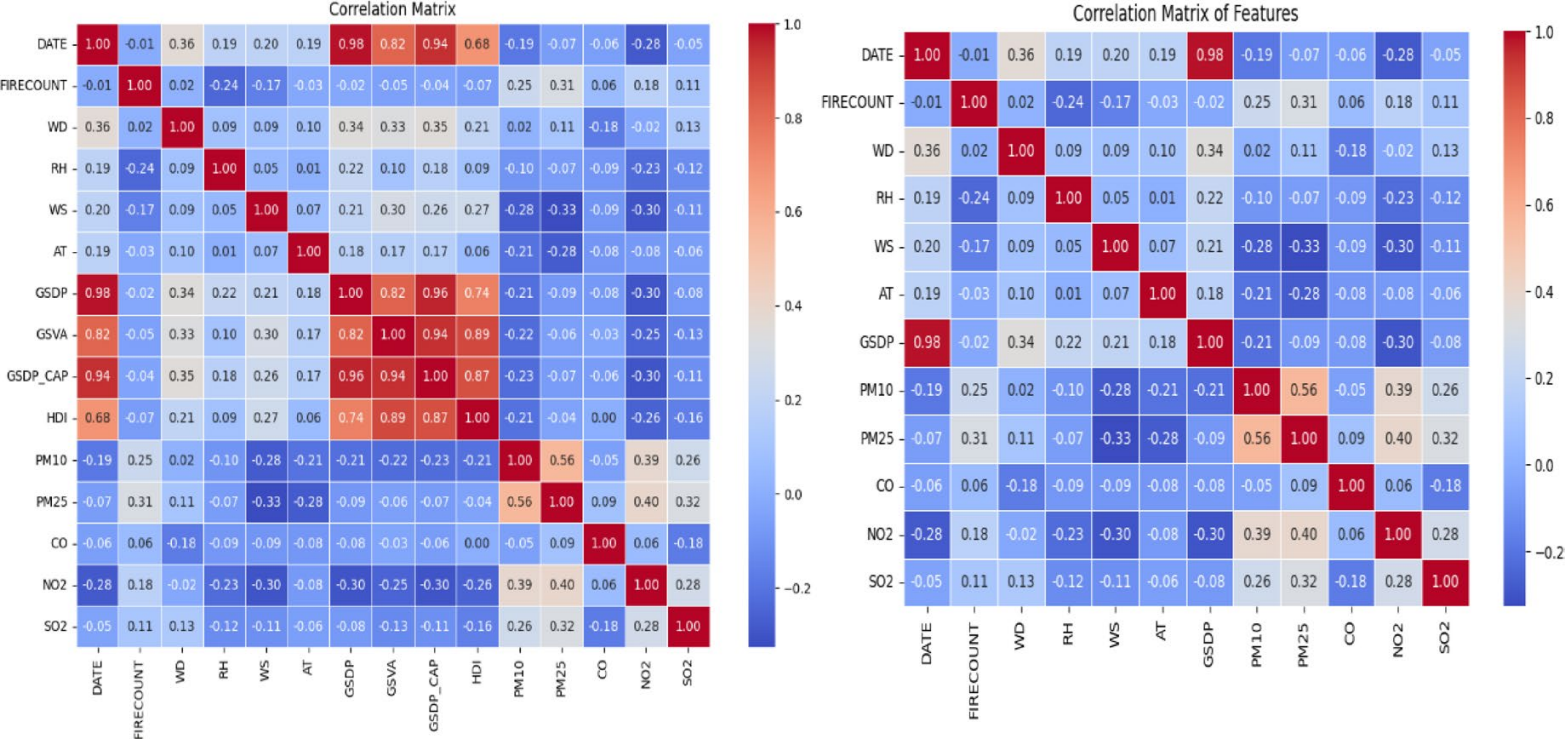
(**a**) Prior removal of redundant features (**b**) After removal of redundant features.

Alongside addressing missing values, it was crucial to assess the significance of each characteristic for the predictive modeling process. At this stage, it was found that several characteristics exhibited slight variation, rendering them redundant and potentially detrimental to the model’s efficiency. For example, economic variables such as GDP, GVA, GDP_CAP, and HDI had stable or nearly stable values throughout the dataset. These variables showed minimal variation, indicating they provided limited information for the model to differentiate between data points. The consistent values in these economic factors could have led to multicollinearity, where the model might overemphasize certain traits, resulting in unstable training and incorrect predictions. Additionally, these traits were less relevant forcasting air quality measures, such as PM10 and *PM*_2.5_, which are more directly influenced by environmental and pollutant-related variables than by economic indicators. As a result, these economic factors were removed from the dataset. Removing unnecessary economic data helped focus the dataset on climatic and pollutant-related features, which have a more direct and dynamic relationship with air quality. Figure 1 shows (a) Before Removing Redundant Features and (b) After Removing Redundant Features. This step reduced the model’s complexity, improving its efficiency and suitability for training.

#### Impact of fire incidents on air pollution: A temporal analysis

**Fig. 2:**
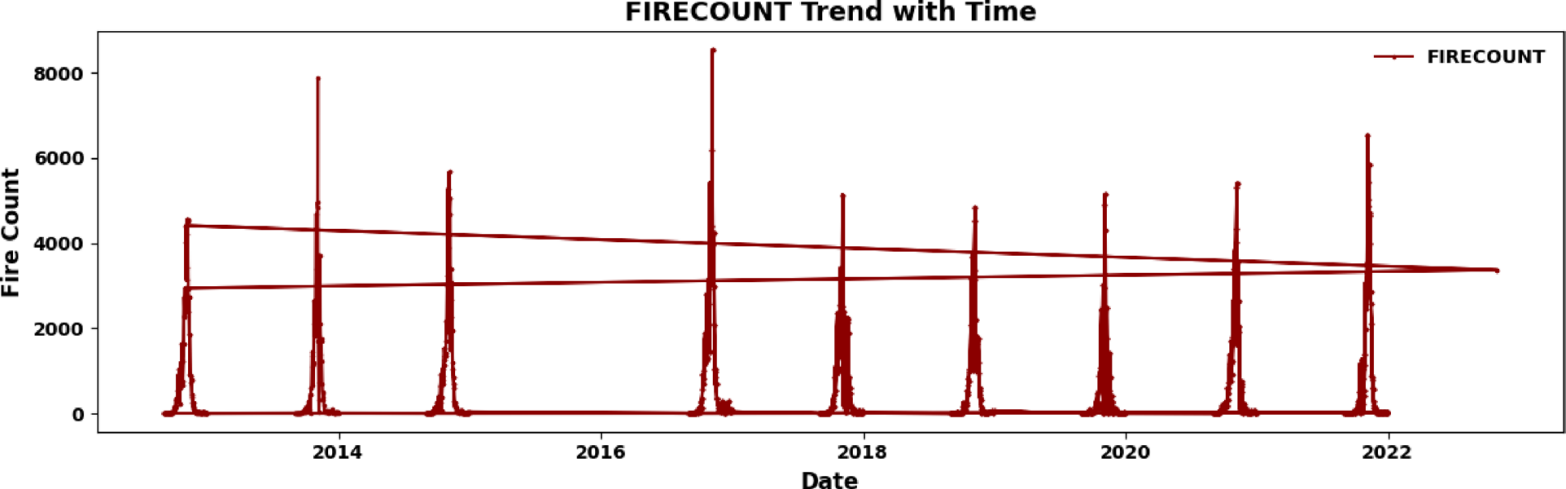
The FIRECOUNT trend from 2012 to 2022 indicates seasonal peaks during post-monsoon stubble burning in Northwest India. Gaps indicate off-season intervals or sporadic constraints on satellite detection.

The visualization in Fig. 2, titled “FIRECOUNT Trend with Time,” shows the daily total of open field fires based on NASA’s VIIRS (Visible Infrared Imaging Radiometer Suite) data from 2012 to 2021. This data focuses on agricultural residue burning in Punjab and Haryana, South Asia, within latitudes 28.90 N to 340 N and longitudes 730 E to 770 E. The information highlights the peak crop residue burning period from September to December. The FIRECOUNT variable, derived from satellite fire detection systems such as MODIS and VIIRS, serves as a region-specific measure indicating biomass combustion events. This study is particularly relevant due to the widespread practice of stubble burning in Punjab and Haryana during the post-monsoon season, which directly impacts *PM*_2.5_ levels in Delhi. Each point on the plot represents the daily fire count during these months, revealing an annual pattern with notable fluctuations. Fire occurrence varies from 1–2 fires per day up to a maximum of 8000, emphasizing the severity of stubble burning in October and November. The trend lines in the graph depict changes in fire counts over the years, indicating seasonal patterns and possible shifts in agricultural practices or regulations. Although the FIRECOUNT variable does not directly measure fire intensity or size, it effectively illustrates trends due to its broad range and inclusion of both small and large fires. Gaps in the data indicate off-season periods or limitations in satellite detection.

**Fig. 3:**
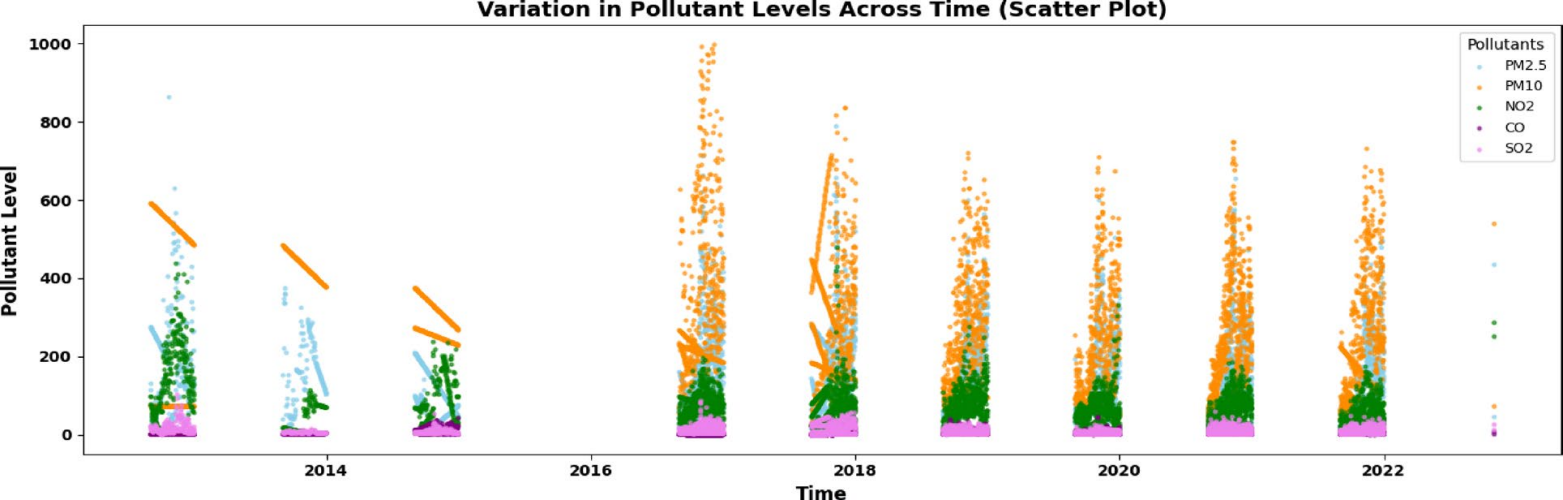
Average temporal trends of five major air pollutants (*PM*_2.5_, PM10, NO2, CO, SO2) across five fixed monitoring stations in New Delhi from 2012 to 2021.

The scatter plot in Fig. 3 displays average patterns over time for five primary air pollutants—*PM*_2.5_, PM10, NO2, CO, and SO2—measured at five stationary monitoring sites in New Delhi from 2012 to 2021. The pollutants were measured in specific units: *PM*_2.5_ and PM10, CO, and SO2. Seasonal trends are evident, particularly the rise in pollution from September to December, primarily due to stubble burning in nearby areas. This seasonal pattern is visible in *PM*_2.5_, PM10, and CO levels. Although NO2 and SO2 show periodic changes, their impact appears smaller. Missing historical data suggests gaps in records; however, the graph clearly shows the impact of human activities, such as industrial emissions, vehicle traffic, and agricultural residue burning, on air quality. These data highlight the seasonal and regional differences in air pollution in New Delhi, offering crucial insights for air quality management and policy development.

**Fig. 4:**
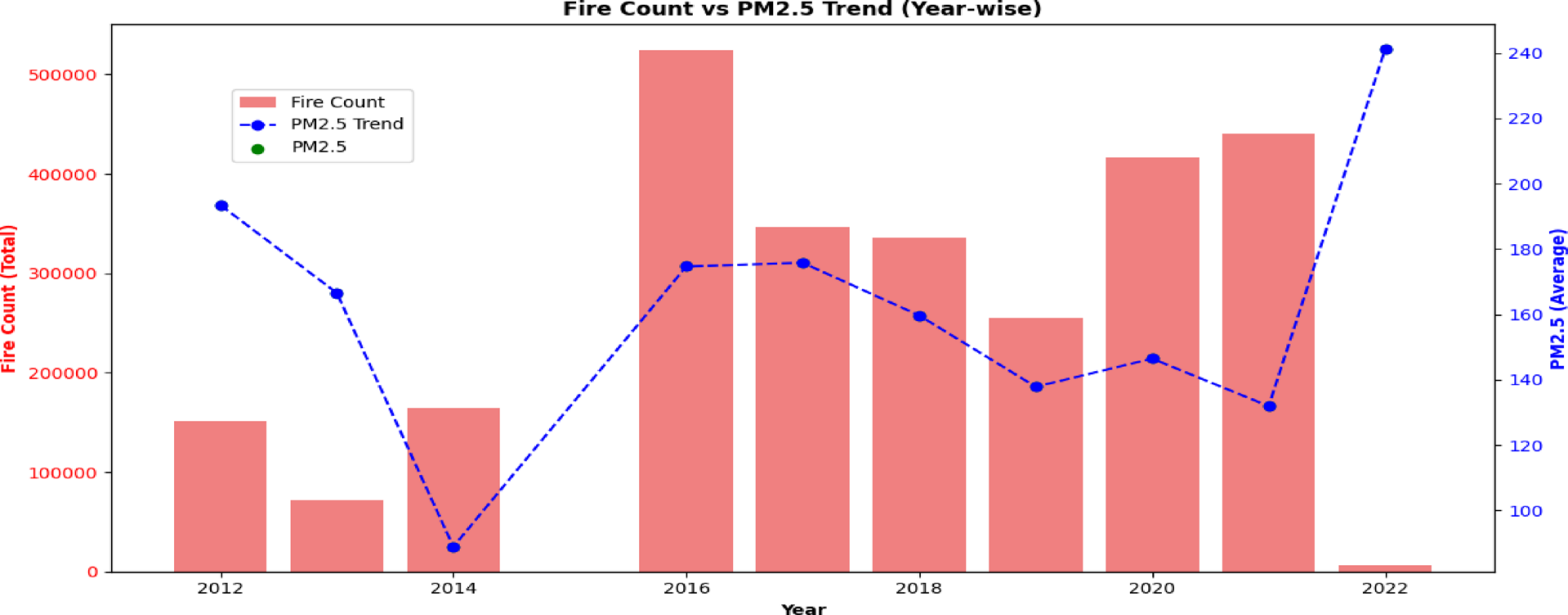
Year-wise trend displaying the link between average *PM*_2.5_ concentration throughout the post-monsoon period (2012–2021, omitting 2015 due to missing data) and seasonal fire activity (FIRECOUNT).

Figure 4 shows the yearly patterns of fire counts and average *PM*_2.5_ levels from 2012 to 2022, illustrating their temporal correlation with a dual-axis format. It excludes 2015 due to insufficient FIRECOUNT data. The visualization aids the study’s objective of predicting air quality by emphasizing the temporal relationship between fire activity and *PM*_2.5_ levels throughout the post-monsoon period (September–December). The blue dashed line and green dots denote average *PM*_2.5_ levels, whilst the pink bars signify total annual fire counts. Significantly, 2014 saw the lowest *PM*_2.5_ levels concurrent with reduced fire activity, whereas 2022 witnessed surges in both, highlighting their robust correlation. Although 2022 demonstrated a clear correlation between intense fire occurrences and elevated *PM*_2.5_ levels, the connection is not entirely linear. From 2016 to 2020, elevated fire counts did not consistently correlate with increased pollution, indicating the impact of additional variables like meteorological conditions, emission regulations, and urban contributions. These findings underscore the necessity of including meteorological and emission factors in prediction models to more accurately represent the intricate dynamics of *PM*_2.5_ pollution. FIRECOUNT and *PM*_2.5_ data were aggregated annually to evaluate their annual correlation, yielding insights into the cumulative effect of biomass combustion on Delhi’s air quality.

#### Temporal -enhanced feature engineering (TEFE)

**Fig. 5:**
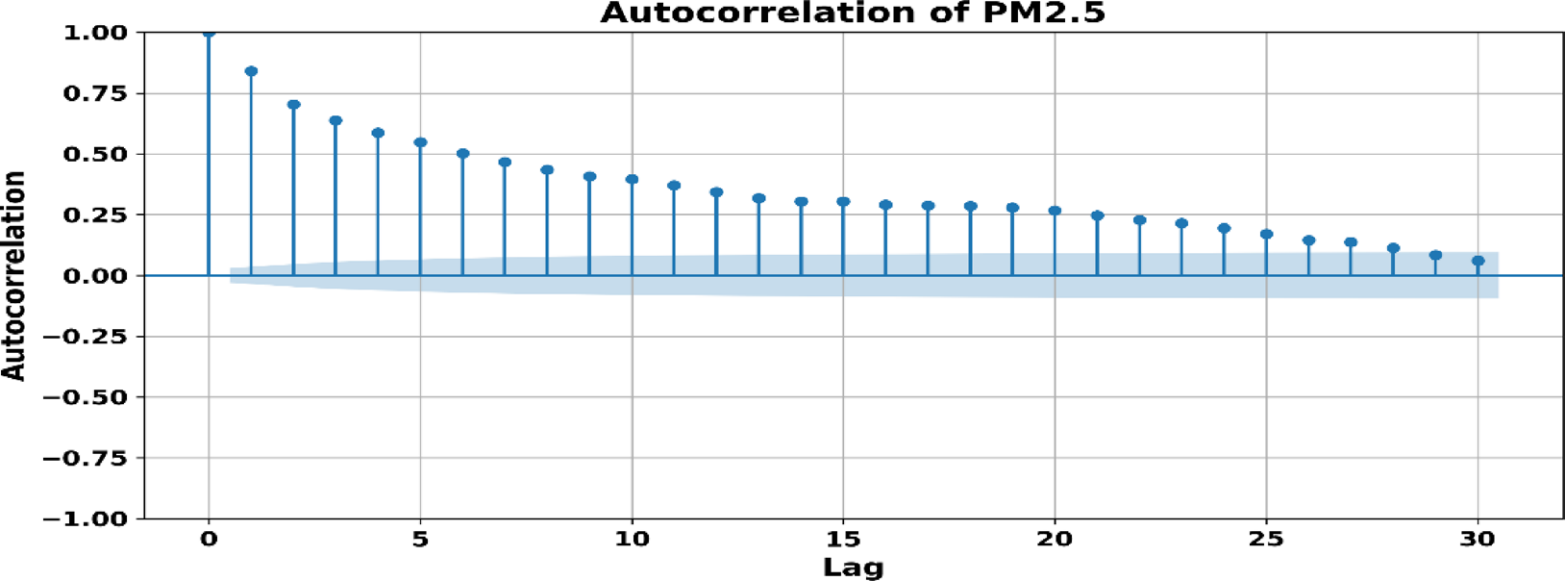
The autocorrelation of *PM*_2.5_ across 30 lags demonstrates significant initial correlations, validating the use of lag characteristics (e.g., lag_1 to lag_3) in the model. The shaded bands represent the 95% confidence interval.

The TEFE technique integrates historical pollutant data, rolling statistics, and temporal factors to elucidate temporal interdependence in *PM*_2.5_ dynamics. Lagged data (e.g., *PM*_2.5__lag_1, lag_2, lag_3) enable the model to assimilate recent historical patterns, whereas the rolling mean and standard deviation over three-day intervals emphasize local variations. Calendar-based attributes, like day-of-week and month, maintain seasonality. An Autocorrelation Function (ACF) study was performed to confirm the incorporation of lagged features as shown in Fig. 5. *PM*_2.5_ demonstrates considerable autocorrelation up to lag 3, hence endorsing the use of short-term lag characteristics in the prediction model. Furthermore, to accurately depict cyclical atmospheric characteristics, Wind Direction (WD), a circular variable was transformed using sine and cosine functions: WD_sin = sin(radians (WD)) and WD_cos = cos(radians(WD)). This encoding maintains angular continuity between 0° and 360°, preventing distortion from linear representations. The original WD column was eliminated after transformation to save repetition. This method improves the model’s capacity to comprehend directional wind patterns pertinent to pollution dispersion.

#### Stationarity testing and treatment

Time series data on air pollution, particularly *PM*_2.5_ and related contaminants, often exhibit non-stationary traits due to seasonal patterns, trends, and external influences such as stubble burning. To verify this, we conducted Augmented Dickey-Fuller (ADF) tests on key pollutant variables, including *PM*_2.5_, PM10, NO2, and SO2. The results indicated that most series were non-stationary at a 95% confidence level, with *p *values exceeding the 0.05 threshold, confirming the presence of unit roots and inherent temporal drift. To address this non-stationarity, this study applied several temporal adjustments during the feature engineering process. Lag variables (*PM*_2.5__lag_1, *PM*_2.5__lag_2, *PM*_2.5__lag_3) and rolling statistics (*PM*_2.5__rolling_mean, *PM*_2.5__rolling_std) were added to the feature set. This helps stabilize trends and highlight small temporal patterns. Additionally, Minmax normalization was used to reduce scale-related differences among all time-dependent features. These preprocessing steps enhance the model’s ability to learn stable representations, thereby improving both convergence and forecasting accuracy under non-stationary conditions.

#### Final dataset preparation

The final dataset included 17 selected characteristics that cover key factors influencing air pollution, such as pollutant levels (*PM*_2.5_, PM10, NO2, CO, SO2), biomass combustion (FIRECOUNT), meteorological variables (Wind Speed, Relative Humidity, Air Temperature), and temporal and historical trends. To forecast short-term *PM*_2.5_ fluctuations, rolling statistics (mean, standard deviation) and lagged values (*PM*_2.5__lag_1, *PM*_2.5__lag_2, *PM*_2.5__lag_3) were used. Weekly and seasonal patterns were represented by the day of the week and the month. Wind Direction was encoded as WD_sin and WD_cos to effectively represent its circular nature without discontinuity.

**Fig. 6:**
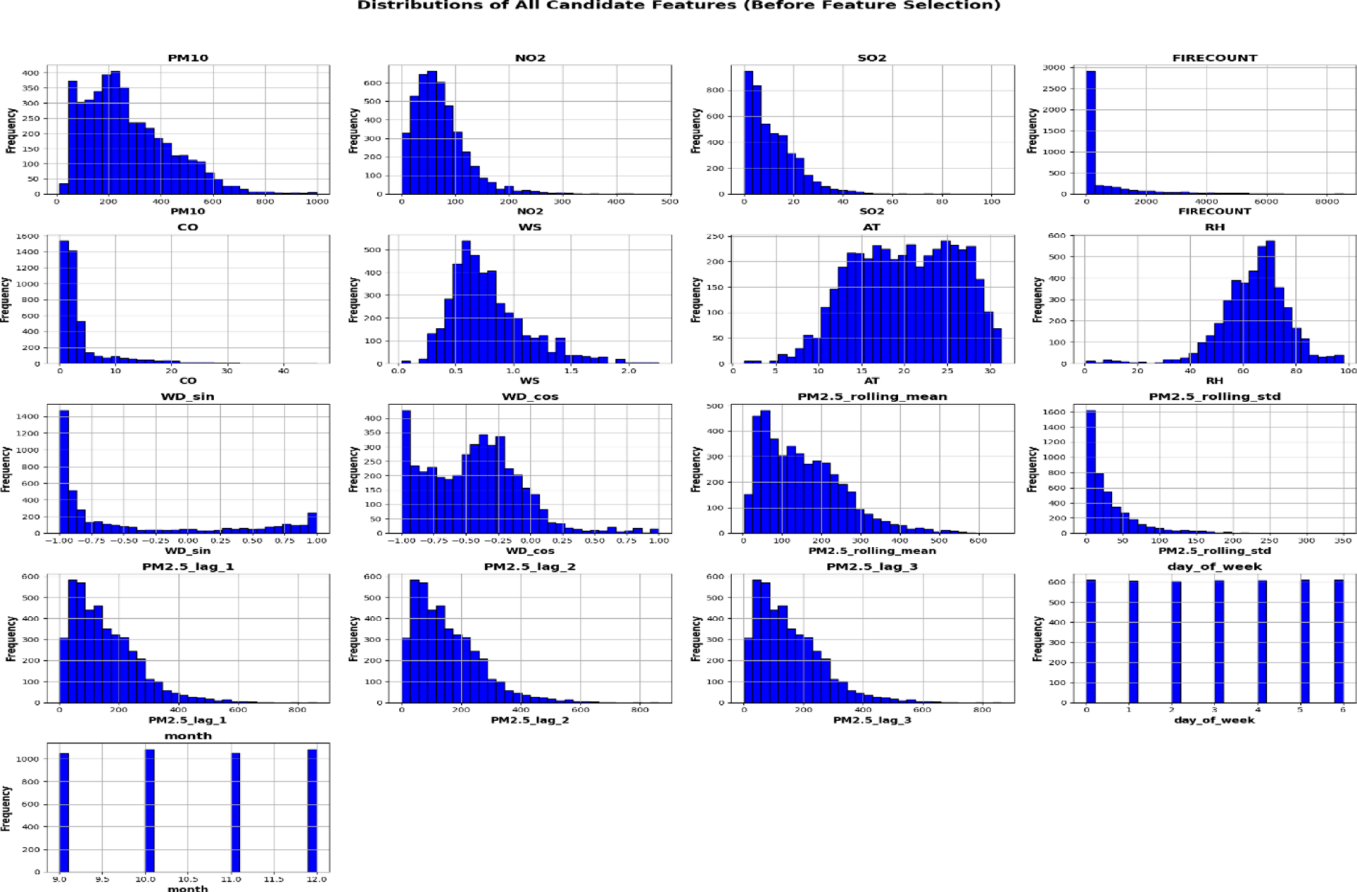
Distribution of features.

Figure 6 shows the distributions of these features, highlighting different patterns: pollutant-related variables and FIRECOUNT have right-skewed distributions with occasional extremes, lagged and rolling *PM*_2.5_ reveal temporal dependencies, while meteorological and temporal features display expected periodic or unimodal patterns. The dataset was cleaned, normalized using Min–Max scaling, and split into training (80%) and testing (20%) sets, providing a solid foundation for accurate *PM*_2.5_ prediction.

#### Scaling and normalization

The dataset was scaled and normalized after removing unnecessary features to prepare it for predictive models. Because of different characteristics on various scales (e.g., PM10 and *PM*_2.5_), the five stages might range from 0 to over a hundred, while Wind Speed (WS) might range from 0 to 20. Therefore, scaling was necessary to prevent any single feature from dominating the learning process due to its size. Min–max scaling was applied to the entire dataset, normalizing each feature to a range between 0 and 1. This normalization ensured that all features contributed equally to the model, allowing the predictive algorithm to process them efficiently. After scaling, the data was validated to confirm there were no abnormalities, and the scaled dataset was saved for further modeling tasks.

#### Feature selection

A hybrid technique integrating Pearson correlation and XGBoost-based significance was employed to guarantee strong and pertinent input characteristics. The Pearson correlation finds variables with robust linear correlations to *PM*_2.5_, whereas XGBoost captures nonlinear dependencies and the cumulative influence of features. This complementary technique guarantees the retention of both directly correlated and significantly important nonlinearly contributing characteristics.

#### Correlation between the features

**Fig. 7:**
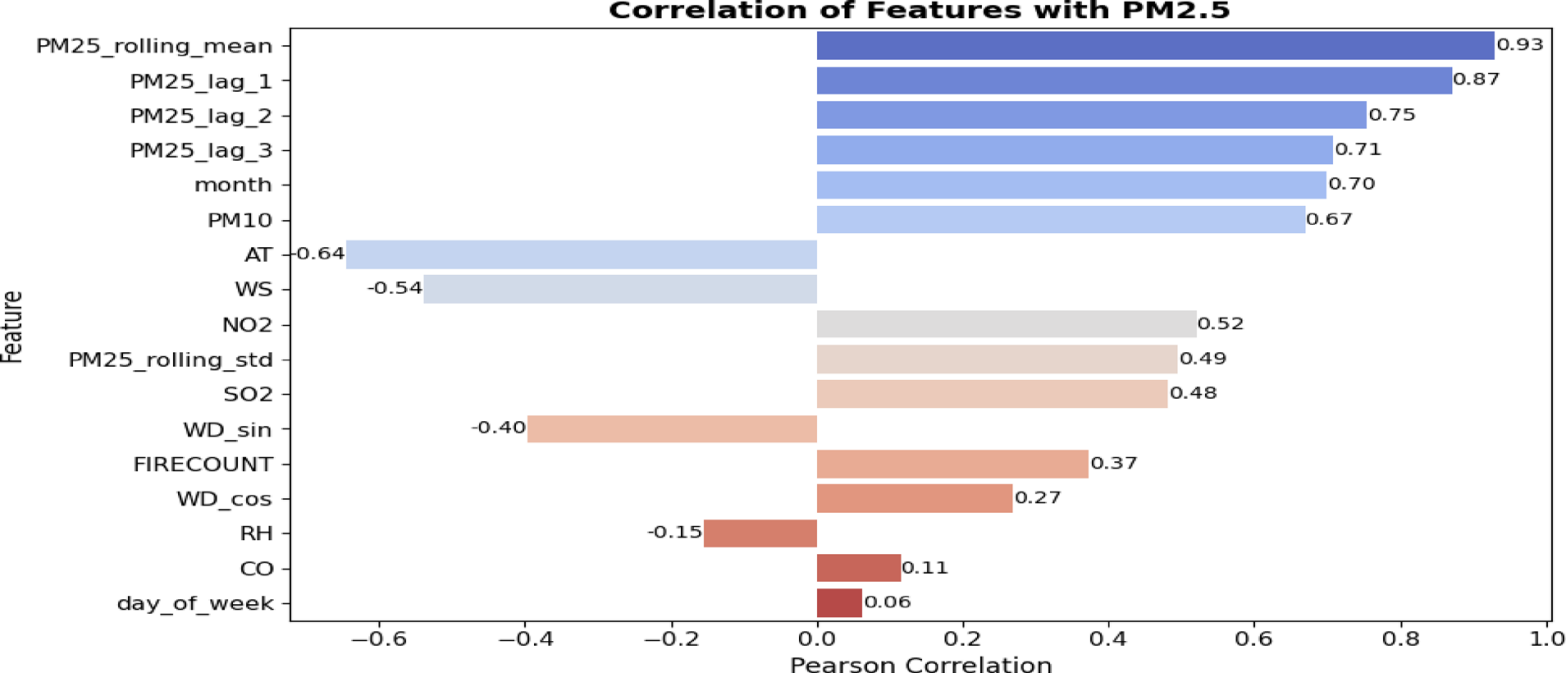
Correlation matrix of features with *PM*_2.5_.

The Pearson correlation coefficient quantifies the strength and direction of linear associations between variables^32^. This study identifies characteristics that are significantly linked with *PM*_2.5_ concentrations for prospective model inclusion. The Pearson correlation study indicates that PM25_rolling_mean (0.93) exhibits the most robust positive association with *PM*_2.5_, highlighting its predictive efficacy. Lagged values *PM*_2.5__lag_1 (0.87), lag_2 (0.75), and lag_3 (0.71) demonstrate robust temporal correlations, affirming the significance of historical patterns. The month (0.70) and PM10 (0.67) further substantiate seasonal and source-related impacts. Moderate to weak associations are noted for NO2 (0.52), *PM*_2.5__rolling_std (0.49), and SO2 (0.48). Meteorological variables such as air temperature (0.64) and wind speed (0.54) have a negative correlation, underscoring their influence on pollution dispersion. Attributes like FIRECOUNT (0.37), WD_sin (–0.40), and WD_cos (0.27) demonstrate restricted linear impact, whereas RH (–0.15), CO (0.11), and day_of_week (0.06) reveal negligible correlation. Figure 7 depicts the correlation matrix of features with *PM*_2.5_; however, some variables may influence outcomes through intricate non-linear interactions, necessitating their assessment using tree-based models like XGBoost.

Given a feature set F = {*f*_1_,*f*_2_,…,*f*_n_), the correlation of each feature *f*_i_ with the target variable *y* is defined as:

1 $$Corr(f_{i} ,{\text{y}}) = \frac{{\sum (f_{i} - \overline{{f_{i} }} ){ }\left( {{\text{ y }} - \overline{ y} } \right){ }}}{{\sqrt {\sum \left( {f_{i} - \overline{{f_{i} }} } \right)^{2} } \sqrt {\sum { }\left( {{\text{ y }} - \overline{ y} } \right)^{2} } }}$$

#### XGBoost regressor for feature relevance scoring

**Fig. 8:**
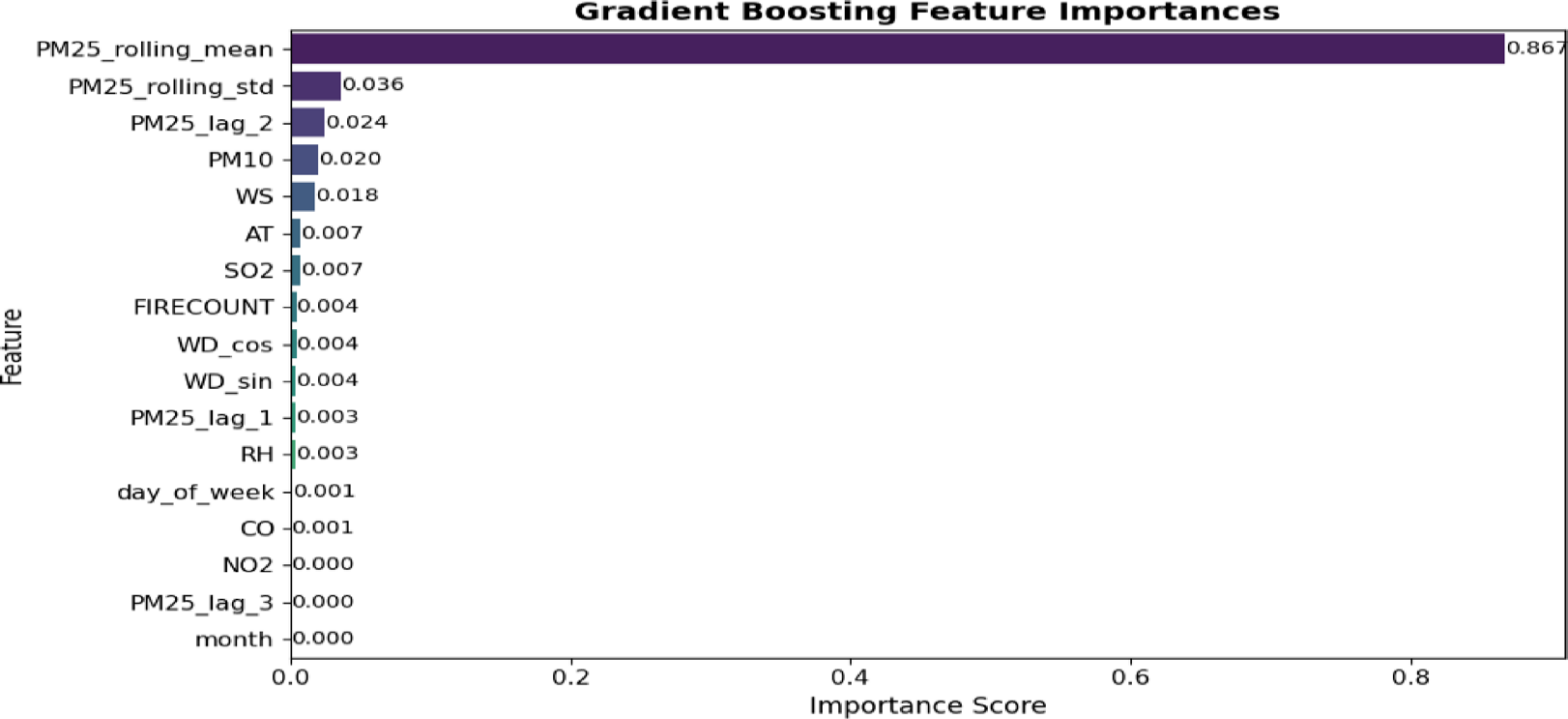
Feature relevance scoring based on XGBoost regressor**.**

Feature selection is essential in air quality prediction as it diminishes the computational cost and improves model accuracy. This study employed the XGBoost Regressor to assess and rank feature significance in predicting *PM*_2.5_ concentrations^33^. The model was developed on an extensive dataset comprising pollutants, meteorological variables, FIRECOUNT, and lagged values. XGBoost assesses feature importance by evaluating their contributions to data splits in decision trees through metrics including gain, frequency, and weight. Table 2 presents the hyperparameter setting of the XGBoost Regressor, and as depicted in Fig. 8, less informative features were systematically removed. The PM25_rolling_mean was identified as the most significant feature, possessing an essential score of 0.867, followed by PM25_rolling_std, PM25_lag_2, and PM10. Conversely, variables such as month, NO2, and CO exhibited minimal scores and were omitted from the final model. This ranking enabled the development of a streamlined, efficient predictive model, enhancing both learning efficiency and generalization. The results were depicted using xgb.plot_importance(), with features ranked according to their contribution scores^34^.

**Table 2:** Hyperparameter setting of the XGBoost regressor.

Hyperparameter	Value	Description
Objective	reg: Squared error	Regression loss function
Colsample_bytree	0.3	Proportion of attributes allocated to each decision tree
Learning_rate	0.1	Step size to weight updates
Max_depth	5	Maximum tree depth
alpha	10	L1 regularization (Lasso)
n_estimators	100	Number of rounds of boosting

Sequentially, XGBoost constructs decision trees, each of which improves predictions by reducing residual errors. Features fi are chosen at each spill to maximize the loss function and ensure better model performance^35,36^. The gain for a specific split is described as

2 $$\left[ {G_{split } = \frac{1 }{{2 }} \cdot \frac{{\left( {G_{{L + G_{R} }} } \right)^{2} }}{{H_{L} + H_{R} + \lambda }} - \frac{{G^{2} L}}{{H_{L } + \lambda }} - \frac{{G^{2} R}}{{H_{R} + \lambda }} } \right] - \gamma$$

where: G_L_,G_R_ are the gradient total for the child nodes on the left and right. H_L_,H_R_ are the sums of Hessians for the left and right child nodes. $\lambda$ is the phrase for regularization that governs complexity. γ is the pruning parameter that makes more splits less acceptable. The tree structure is optimized utilizing gain-based feature significance,

3 $$I(f_{i} ) = \mathop \sum \limits_{t = 1}^{T} G(f_{i } ,t)$$

where *G(f*_*i*_*,t)* is the gain rate of feature* f*_*i*_ in tree t.

#### CorrXGBoost-rank: a fusion-based feature selection algorithm integrating correlation analysis and XGBoost feature importance

**Fig. 9:**
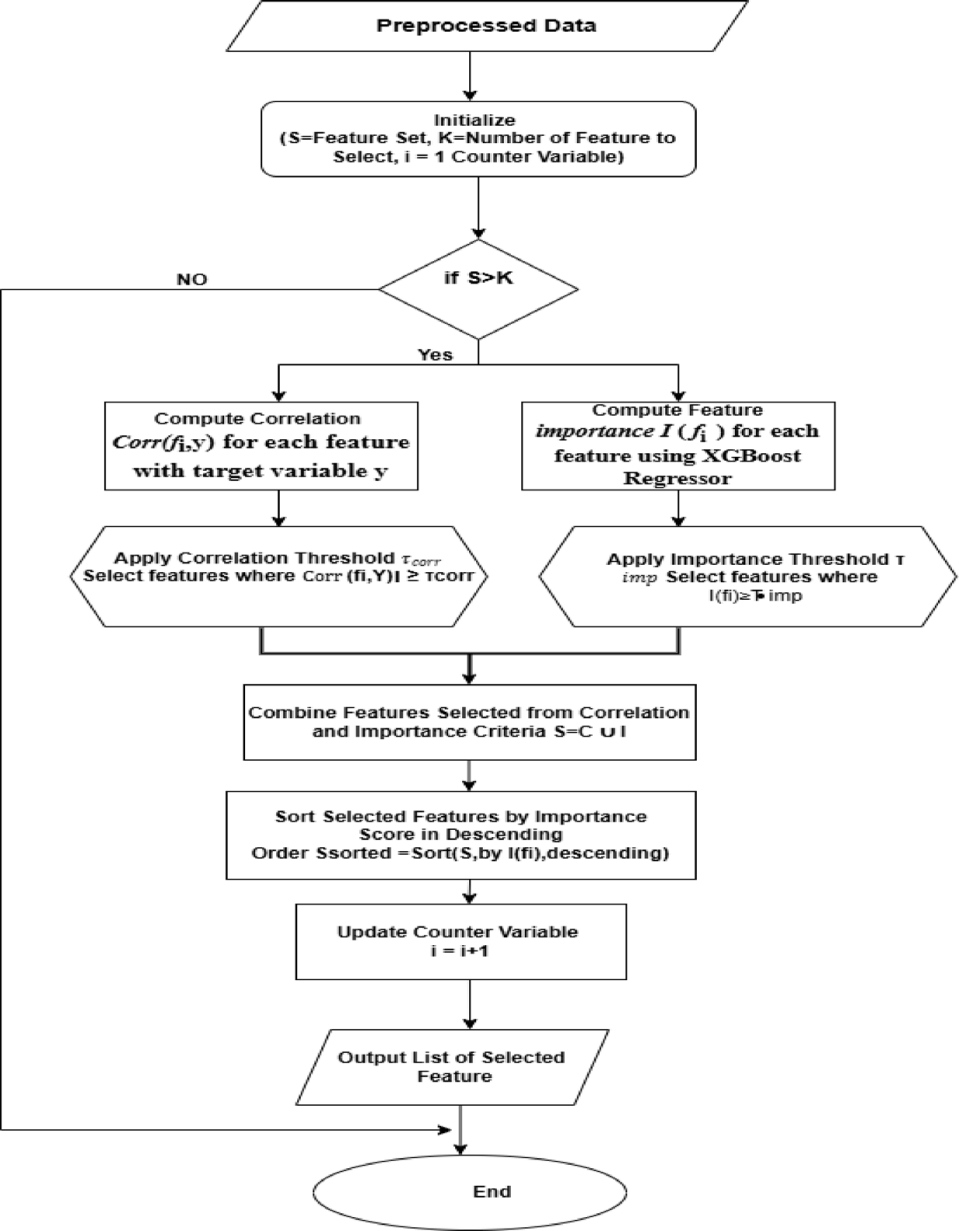
Flowchart for significant feature selection using CorrXGBoost-rank.

This study proposed CorrXG-Rank, a hybrid feature selection approach that combines Pearson correlation analysis with XGBoost-based importance ranking to improve the reliability and efficiency of air quality forecasting. The objective is to preserve features that demonstrate either significant linear correlations with the target variable or have substantial nonlinear effects on predictive efficacy. Figure 9 and Table 3 show the Flowchart and pseudocode for significant feature selection using CorrXGBoost-Rank.

**Table. 3:**
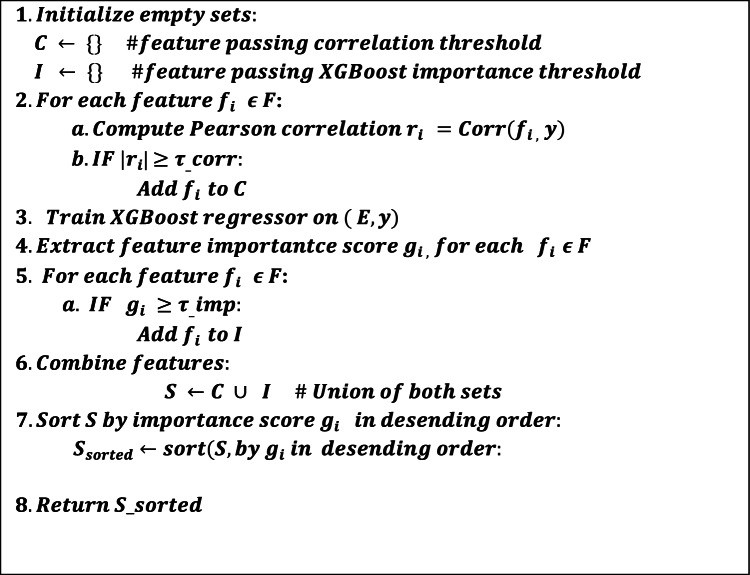
Pseudocode for CorrXGBoost-Rank: a hybrid feature selection approach integrating correlation and XGBoost importance.

Features were selected based on a correlation threshold of |r|≥ 0.30 or an XGBoost importance score of ≥ 0.015. This dual-criteria methodology guarantees the incorporation of variables that are both statistically significant and influential within a tree-based learning framework. The thresholds (τ = 0.30 for correlation and γ = 0.015 for XGBoost importance) were not chosen arbitrarily. Still, they were empirically optimized via grid search across τ ∈ [0.1, 0.5] and γ ∈ [0.005, 0.03], to minimize MAE and RMSE while maximizing R^2^ on the validation set. This hybrid selection strategy guarantees the incorporation of both statistically significant and model-influential features. Table 4 displays the chosen features alongside their correlation coefficients, XGBoost importance scores, and the criteria they fulfilled.

**Table 4:** Feature selection utilizing Pearson correlation (|r|≥ 0.30) and XGBoost importance (≥ 0.015). Features that met either criterion were chosen for model training.

No of feature	Selected features	Correlation (|r|)	XGB importance score	Pass corr > = 0.30	Pass XGB > = 0.015	Selected
1	PM25_rolling_mean	0.93	0.867	True	True	True
2	PM25_lag_1	0.87	0.003	True	False	True
3	PM25_lag_2	0.75	0.024	True	True	True
4	PM25_lag_3	0.71	0	True	False	True
5	Month	0.7	0	True	False	True
6	PM10	0.67	0.02	True	True	True
7	AT	0.64	0.007	True	False	True
8	WS	0.54	0.018	True	True	True
9	NO2	0.52	0	True	False	True
10	PM25_rolling_std	0.49	0.036	True	True	True
11	SO2	0.48	0.007	True	False	True
12	WD_sin	0.4	0.004	True	False	True
13	FIRECOUNT	0.37	0.004	True	False	True

For example, *PM*_2.5__rolling_mean, PM10, and *PM*_2.5__rolling_std satisfied both criteria, whereas features like *PM*_2.5__lag_1 and AT were preserved due to their strong correlation despite inferior XGBoost scores. In contrast, certain variables exhibiting modest correlation yet significant relevance in tree-based models (e.g., *PM*_2.5__lag_2, WS) were also chosen. Thirteen features were retained from a total of seventeen, resulting in a concise yet informative input space. This fusion methodology offers a balanced compromise between statistical interpretability and predictive efficacy, enhancing the model’s accuracy and generalization ability.

This study integrates the CorrXGBoost-Rank feature selection method with the TL-LSTM-MHA deep learning model. CorrXGBoost-Rank effectively filters and ranks features according to statistical correlation and model-driven significance. The chosen features are subsequently utilized by the transfer learning-based LSTM with Multi-Head Attention, facilitating superior temporal pattern recognition while minimizing computational complexity and enhancing generalization during periods of elevated pollution.

### Methods

The fundamental principles serve to construct the theoretical framework for this investigation and guide the formulation of the suggested methodology within this segment.

#### Definition of transfer learning

An approach to machine learning known as transfer learning (TL) uses information from a source domain or activity to improve efficiency in an associated target domain or activity. It speeds up model training, lowers computing costs, and tackles issues like a lack of labeled data.TL is especially helpful for sequential data, like air pollution time series, because patterns from one context may be transferred to another. It entails prior training with a large dataset and fine-tuning over a smaller, task-specific dataset. TL is useful for managing intricate univariate time series data in air quality prediction, reducing the requirement for large, labeled datasets and processing resources. It makes it possible to customize models pre-trained on large datasets to pollutants or geographical areas, providing shorter training times, better generalization, efficient management of data scarcity, and higher accuracy for prediction in applications such as *PM*_2.5_ forecasting^9^.

In machine learning, along with deep learning, transfer learning is the process of applying information from one problem’s solution to another that is similar but distinct^37^. Because the pre-trained model may use characteristics learned in the source domain, this method works especially well when the destination domain has less data. Let $DS= XS, P(XS)$ indicate the source domain, where *X*_*S*_ represents the feature space and *P(X*_*S*_*)* denotes the marginal probability distribution. Similarly, the source task is defined as TS = {Y_S_, f_S_ (X_S_)}, where YS is the label space and f_S_ is the predictive function. In transfer learning, the objective is to transfer knowledge from *(D*_*S*_*, T*_*S*_*)* to the target domain *(D*_*T*_*, T*_*T*_*),* where *D*_*T*_ = *{X*_*T*_*,P(X*_*T*_*)}* and *T*_*T*_ = *{Y*_*T*_*, F*_*T*_*(X*_*T*_*)}*, under the condition that *D*_*S*_ $\ne$ *D*_*T*_ or *T*_*S*_ $=$ *T*_*T*_^28,38^. This work employs transfer learning through the pre-training of a deep LSTM-MHA model, thereafter, fine-tuning it on the Delhi-specific *PM*_2.5_ dataset, as detailed in Sect. 3.4.

#### LSTM based architecture

**Fig. 10:**
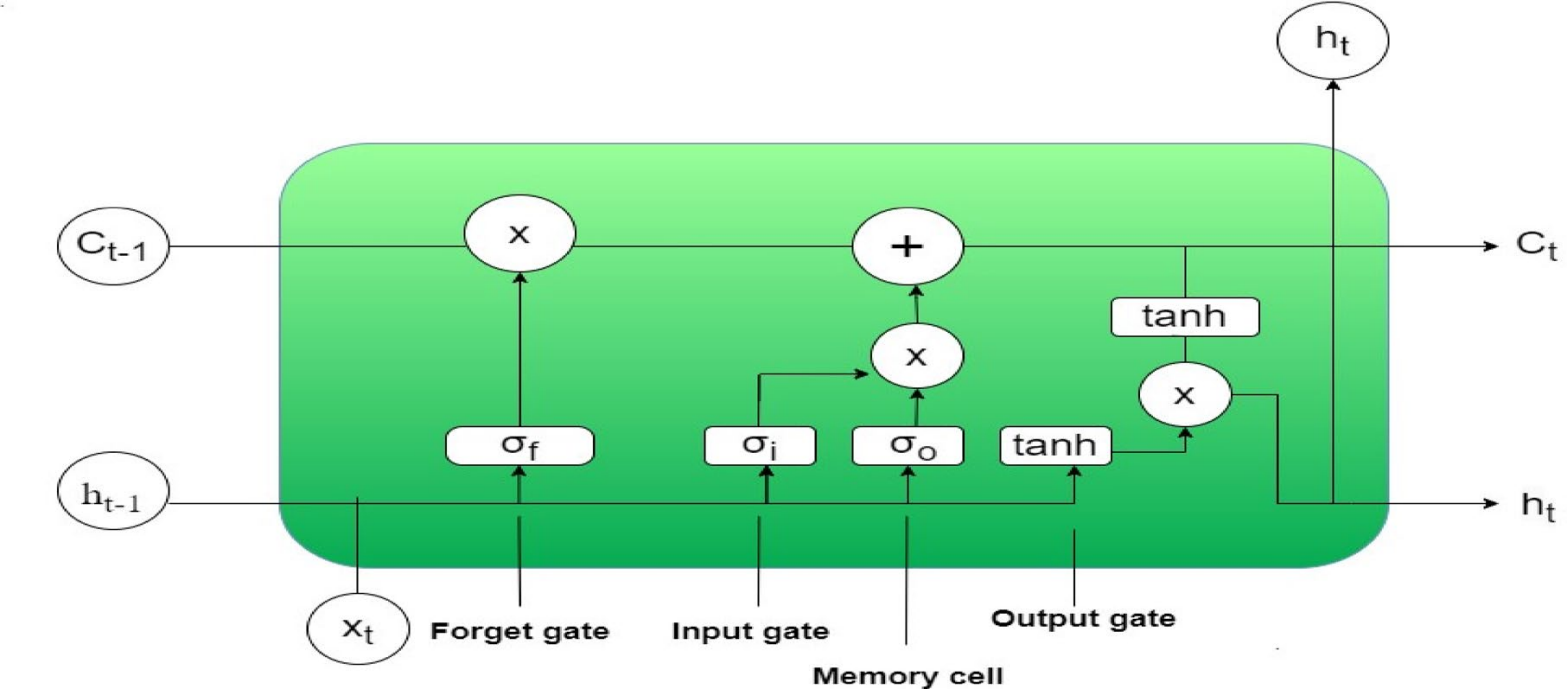
Internal Structure of LSTM

Establishing long-term links between states in deep Recurrent Neural Networks (RNNs) is empirically challenging due to the gradient vanishing issue. A set of gates is incorporated into the LSTM network, which is a modified RNN design, to control information flow. This method effectively identifies the gradient vanishing issue in RNNs^39^. By replacing traditionally hidden neurons with memory units that can store and retrieve information, the LSTM design enables the system to accurately reliance. The input gate, forget gate and output gate are the three kinds of gates that make up the memory block. The gates manage the flow of information into and out of the cell^40,41,42^. The following describes the main LSTM calculating equation. The input is denoted by X_t_. C_t−1_ and h_t−1_ are the parameters that the previous LSTM supplied. The input gate, forget gate and output gate are indicated by the parameters i_t_, f_t_, and o_t_ respectively. The internal construction of the LSTM is shown in Fig. 10.

In this research, initially, the source domain is used to train a pre-trained LSTM model that incorporates Multi-Head Attention (MHA). The LSTM layer processes sequences that identify temporal dependencies, and the results are computed as follows,

4 $$i_{t } = \sigma ( W_{x }^{i} x_{t} \, + \,W_{h}^{i} \,h_{t - 1} \, + \,W_{c }^{i} o\,C_{t - 1} + b_{i} )$$

5 $$f_{t} = \sigma (W_{x }^{f} x_{t} \, + \,W_{h}^{f} \,h_{t - 1} \, + \,W_{c }^{f} \,o\,C_{t - 1} + b_{f} )$$

6 $$C_{t } = f_{t} o C_{t - 1 } \, + \,i_{t} \,o\,\tanh \,W_{x }^{c} x_{t} \, + \,W_{h}^{c} \,h_{t - 1} + b_{c} )$$

7 $$O_{t} \, = \,\sigma ( W_{x }^{o} x_{t} \, + \,W_{h}^{o} \,h_{t - 1} \, + \,W_{c }^{o} \,o\,C_{t - 1} + b_{o} )$$

8 $$h_{t} = O_{t} o\;\tanh (C_{t} )\;{\text{or}}\;h_{t } = \sigma (W_{h} h_{t - 1} + W_{x} x_{t} + b_{h} )$$

#### Multi-head attention mechanism

The multi-head attention mechanism is a sophisticated method for scaling dot-product attention. It allows the model to learn numerous connections by using several description subspaces^24,43^. This is the way it works. Linear transformations are performed on the input variables Quary(Q), Key (K), and Value (V) for each model^44^. The modified parts are evaluated in parallel across attention heads, generating outcomes of dimensionality d_v._ The result is generated by concatenating the output from all h heads via the Concat function and applying an extra linear modification. This method enables the model to record more varied associations across multiple subspaces, boosting its capacity to handle data. Despite the use of several heads, the total computational complexity is comparable to a single-head attention layer whilst each head acts on a decreased dimensionality^45,46^. The calculations are outlined below:

Construct the scaled dot product score:

9 $$S_{i} { }\left( {{\text{Q }},K_{i} } \right){ } = { }\frac{{K_{i}^{T} {\text{Q}}}}{{\sqrt {d_{k} } }}$$

Employ the softmax function to normalize the scores:

10 $$\alpha_{{i{ }}}^{s} = { }\frac{{exp{ }\left( {S_{i} } \right)}}{{\mathop \sum \nolimits_{i = 1}^{n} exp(score\left( {S_{i,} } \right)}}$$

11 $${\text{Attention }}\left( {{\text{Q}},{\text{ K}}_{{{\text{i}},}} {\text{V}}_{{\text{i}}} } \right) \, = {\text{ Softmax}}(\,\frac{{K_{i}^{T} {\text{Q}}}}{{\sqrt {d_{k} } }}) \cdot {\text{V}}_{{\text{i}}}$$

12 $${\text{Multihead}}\,\left( {{\text{Q}},{\text{ K}},{\text{ V}}} \right)\, = \,{\text{Concat}}\,\left( {{\text{head}}_{{{1},}} {\text{head}}_{{\text{n}}} } \right)\, \cdot \,{\text{W}}^{{\text{o}}}$$

where head_i_ = Attention (Q W_i_^Q^, KW_i_^K^,V W_i_^V^) are trainable parameters.

**Fig. 11:**
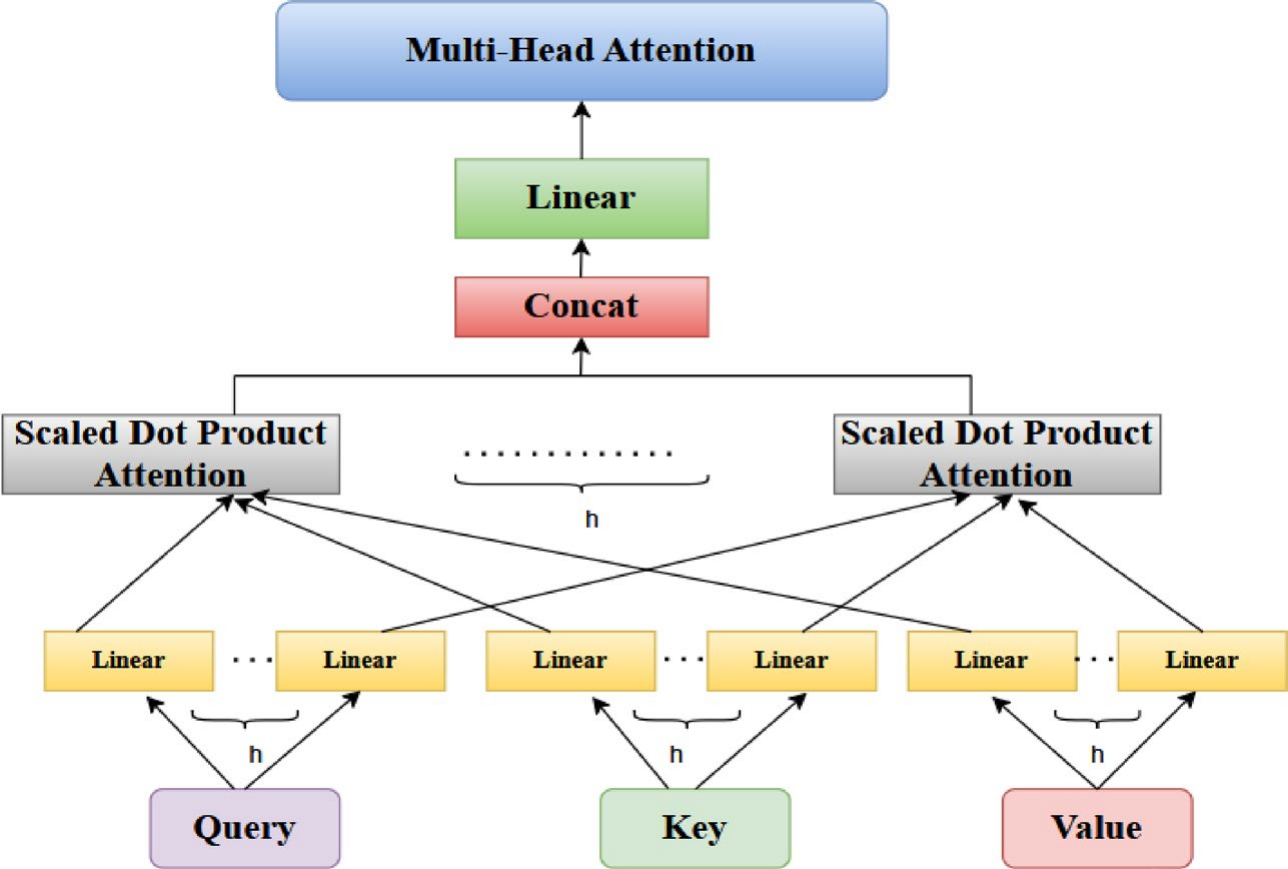
The structural design of the Multi-Head Attention Mechanism.

This work uses 4 simultaneous attention heads (h = 4), each operating on an optimized dimensionality (d_k_ = d_v _= $\frac{\text{dmodel}}{h}$ = 64). This split enhances the model’s capacity to obtain broad and varied information while maintaining computational effectiveness, enabling multi-head attention^47^ that is cost-comparable to single-head attention. Figure 11 displays the structural design of the Multi-Head Attention Mechanism.

#### Integrated TL-LSTM-MHA modelling framework

The preceding sections outline the essential components Transfer Learning (TL), Long Short-Term Memory (LSTM), and Multi-Head Attention (MHA)—included in this study to develop a cohesive air quality prediction model. The suggested TL-LSTM-MHA structure integrates these components in a progressive manner. An initial LSTM-MHA model is trained on historical air quality data augmented with temporal and environmental variables. The LSTM layer captures sequential relationships in pollutant levels by processing data in temporal order, thereby preserving time-step alignment. As a result, the MHA layer enhances the model’s ability to focus on important temporal steps and feature interactions by distributing attention across multiple heads.

**Fig. 12:**
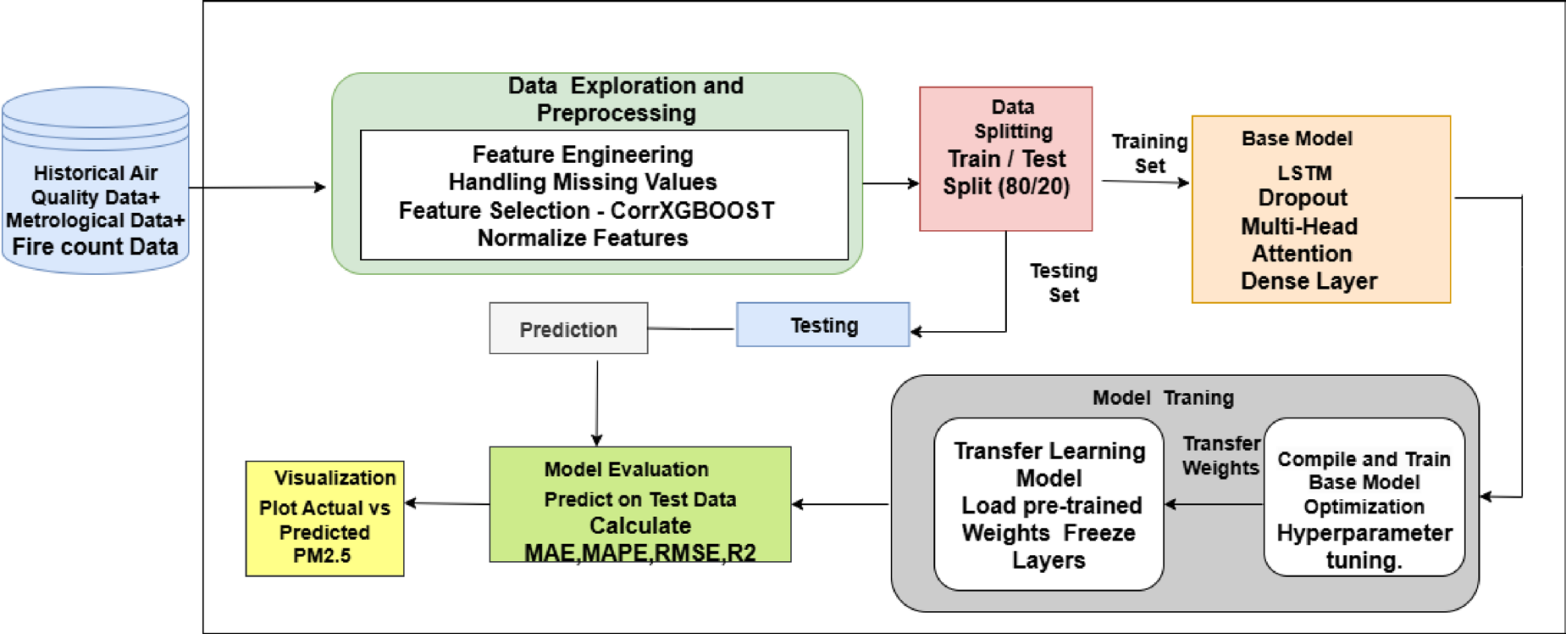
Architecture diagram of proposed system.

The function of LSTM before the MHA is crucial: it encodes the input sequence into a temporally consistent representation, enabling MHA to work efficiently without needing explicit positional encoding. This naturally preserves the sequence order. While positional encoding is usually used in transformer systems, it was deemed unnecessary here because the LSTM already captured the sequential context. The number of attention heads in the MHA was set at four, balancing model complexity and performance. The choice of four was based on previous studies showing effective results in similar time-series tasks and computational efficiency. However, a thorough search for the optimal number of heads was not performed. Future work may include a detailed evaluation to further improve this design. Transfer learning uses pre-trained weights from a base model to initialize a new model. The early layers are frozen to retain previously learned representations, while the later layers are fine-tuned with domain-specific data. This combined approach allows the model to leverage existing patterns, adapt to the unique characteristics of Delhi’s air quality, and deliver reliable, generalizable predictions. Figure 12 in Section “Model development and evaluation” illustrates the whole pipeline.

## Experimental details

The experimental specifics encompass the data splitting methodology, assessment measures, and model-building protocols. Performance was evaluated using key measures including MAE, RMSE, and R^2^. The TL-LSTM-MHA model was executed with established training techniques and refined by tenfold cross-validation to guarantee robustness and generalizability.

### Experimental setting

The research was conducted using a Windows 10 operating system with an Intel i5-8400 processor running at 2.80 GHz, along with an NVIDIA GeForce GTX1060 graphics card with 5 GB of memory and 24 GB of RAM. Data manipulation, prototype development, and operational setup were carried out using the Python 3.6 environment, which utilized numerous open-source libraries and frameworks, including Pandas, NumPy, and PyTorch^25,42^. Our study focuses on a large dataset comprising 4270 entries with 25 distinct attributes. The training set includes 3416 instances (80%), while the validation set contains 854 instances (20%).

### Performance metric

The efficacy of the optimal designs is evaluated using three different criteria: Mean Absolute Error (MAE), Root Mean Square Error (RMSE), and the Coefficient of Determination (R2)^48^.

13 $$MAE = \frac{1}{n} \mathop \sum \limits_{i = 1}^{n} (y_{i - } y_{i }{\prime} )$$

14 $$RMSE\, = \,\sqrt {\frac{1}{n} \mathop \sum \limits_{i = 1}^{n} (y_{i - } y_{i }{\prime} )^{2} }$$

15 $$R^{2} = 1 - \frac{{ \mathop \sum \nolimits_{i = 1}^{n} (y_{i - } y_{i }{\prime} )^{2} }}{{ \mathop \sum \nolimits_{i = 1}^{n} (y_{i - } y_{i }{\prime} )^{2} }}$$

The symbol indicates the estimated rate of the element while showing the actual rate for a specific sample. The variable n refers to the total number of elements. Lower MAE, and RMSE values suggest better prediction accuracy. Conversely, higher R2 values indicate a better fit of the model. The R2 value ranges from 0 to 1, with values closer to 1 indicating more accurate predictions.

### Model development and evaluation

Training and evaluation in the field of deep learning are vital steps for developing and refining models that perform tasks effectively. The training dataset, which accounts for 80% of the total data, is used to train the model, while the remaining 20% is reserved for testing. Key features were identified through domain expertise and mutual information scores. After assessing the relevance of each feature, those deemed irrelevant or with minimal impact were removed from the dataset.

**Fig. 13:**
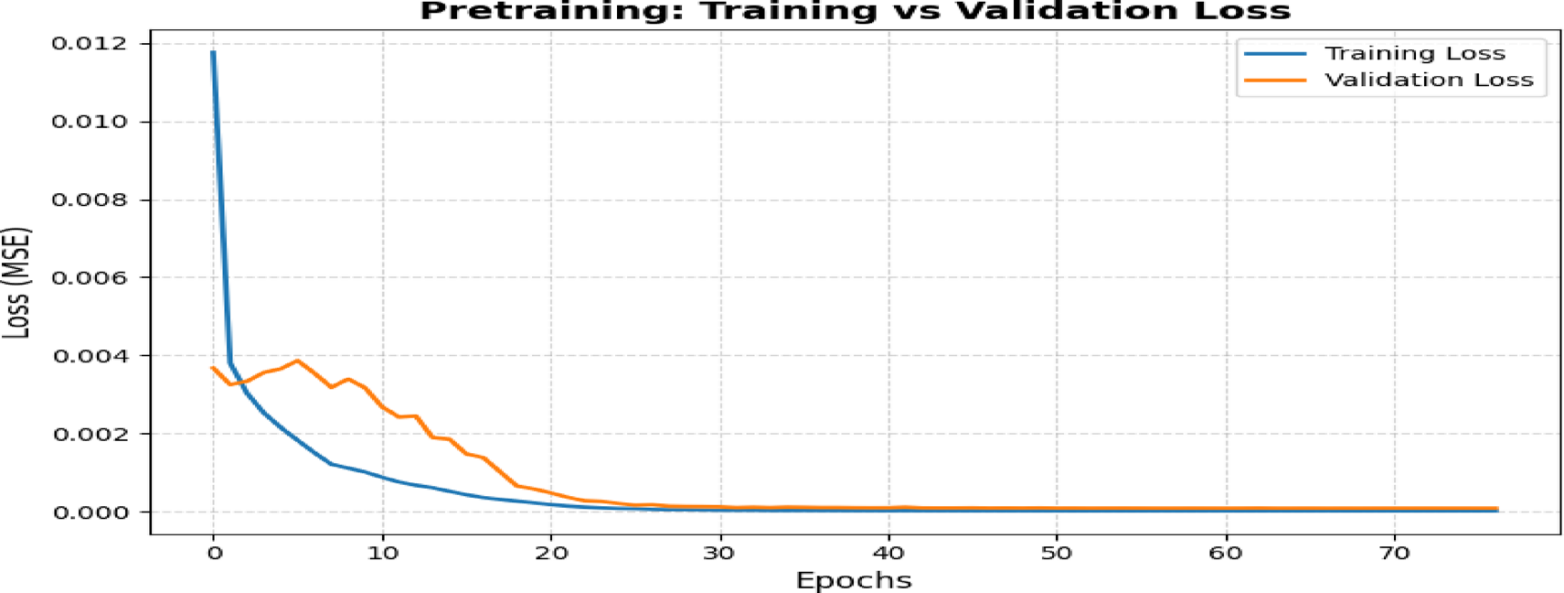
Comparison of training and validation losses over epochs.

**Fig. 14:**
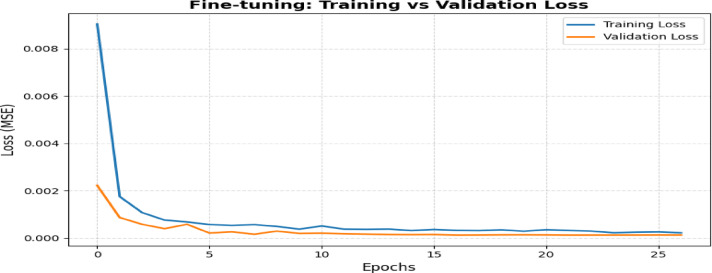
Depicts the training and validation loss curves throughout pretraining.

The model’s evolution begins with establishing the Base Model, which combines Long Short-Term Memory (LSTM) with Multi-Head Attention (MHA). Figure 12 shows the architecture diagram of the proposed system. The correlation criterion τ = 0.30 and the XGBoost importance threshold γ = 0.015 were experimentally determined to eliminate weak and less relevant features. Grid search studies including combinations of (τ, γ) (e.g., τ ∈ [0.1, 0.5], γ ∈ [0.005, 0.03]) validated that these thresholds attained near-optimal MAE, RMSE, and R^2^ on the target test set (refer to Fig. 16). A grid search method was used with fivefold cross-validation to find the best configuration of the TL-LSTM-MHA model. The hyperparameter search space was based on existing research and established techniques in time-series deep learning. The following parameters were tested: LSTM units [64, 100, 128], dropout rates [0.3, 0.4, 0.5], attention heads^2,4,8^, key dimensions for multi-head attention [32, 64], learning rates [1e−3, 5e−4, 1e−4], and batch sizes [16, 32, 64]. The input layer was optimally set up to receive reshaped data, making it ready for the LSTM layer, which analyzes temporal relationships using 100 units with ReLU activation. A Dropout layer with a rate of 0.4 is included to reduce overfitting. The MHA layer, consisting of 4 heads and a key dimension of 64, extracts essential features by focusing on different segments of the input sequence. The outputs from the LSTM and MHA layers are combined and then passed through a Dense layer with 64 units that refine the learned features, culminating in a final output layer using linear activation for regression. The model uses Mean Squared Error as the loss function; Table 5 delineates the conclusive model architecture, compilation parameters, training configuration, and evaluation metrics, all chosen according to the optimal hyperparameters determined via grid search, and Fig. 13 shows the comparison of training and validation losses over epochs. Figure 14 depicts the training and validation loss curves throughout pretraining. The foundational model was trained on the source domain (historical data), reaching convergence in approximately 30 to 40 epochs without overfitting. The fine-tuning phase, performed on the target domain (post-2021 data), used pretrained weights and achieved convergence in about 10 epochs with consistently low training and validation losses (Fig. 14). Early stopping (patience = 10) and learning rate reduction (factor = 0.5, patience = 5) were applied to prevent overfitting and speed up convergence.

**Table 5:** Model’s architecture, compilation, training, and evaluation metrics.

Hyperparameter	Setting
Model type	LSTM + Multi-head attention
LSTM units	100
LSTM activation function	ReLU
Dropout rate	0.4
Input shape	(1, num_features)
Multi-head attention	4 heads, key dimension = 64
Concatenation	[LSTM_output, MHA_output]
Dense layer units	64
Optimizer	Nadam
Learning rate	0.0005
Loss function	MSE
Early stopping patience	10 epochs
Learning rate reduction patience	5 epochs
Learning rate reduction factor	0.5
Minimum learning rate	1e-6
Batch size	32
Epochs	100
Validation split	0.2
Pretraining domain	Source (data before 2021)
Fine-tuning domain	Target (data from 2021 onward)
Transfer learning layer freezing	First 3 layers
Transfer learning optimizer	Nadam

**Fig. 15:**
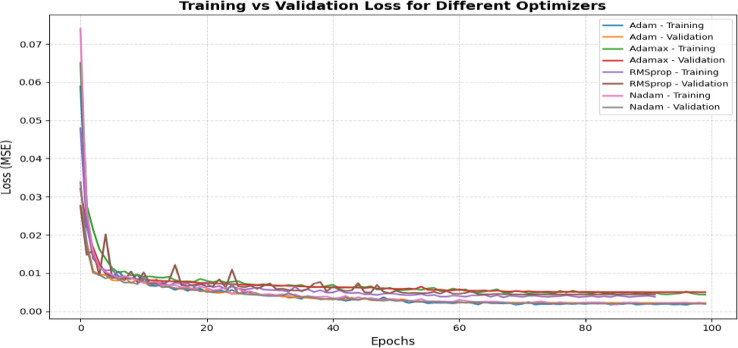
Training vs validation loss with different optimizers.

Figure 15 displays the training vs validation loss with different optimizers. The NADAM optimizer was chosen for optimization because of its exceptional performance regarding RMSE and R2 scores. NADAM integrates the advantages of ADAM with Nesterov’s momentum, offering superior convergence and stability in training relative to other optimizers such as AdamW and RMSprop. This led to a more precise model for forecasting *PM*_2.5_ concentrations, establishing NADAM as the preferred optimizer.

The suggested transfer learning methodology was pre-trained on the source domain (data before 2021) and subsequently fine-tuned and assessed on the target domain (data from 2021 onwards) using the experimental framework (source_df =  < 2021, target_df =  >  = 2021). A model with the same architecture was created and initialized using weights acquired from the source domain.

The initial three layers were frozen during fine-tuning to maintain the overarching temporal patterns acquired from the source. The residual layers were trained on the target data to acclimatize to its seasonal and contemporary attributes. This method, employing the same loss function (MSE) and optimizer (Nadam), expedited convergence, reduced the required target domain data, and enhanced generalization by utilizing past information for resilient *PM*_2.5_ prediction during the target time.

The combination of LSTM with Multi-Head Attention enables the model to understand both temporal dependencies and complex long-range connections, thereby improving its accuracy in *PM*_2.5_ forecasting.

This work incorporates Multi-Head Attention (MHA) in parallel with LSTM to maintain temporal alignment while preserving the sequential structure of the input data. The outputs of both layers are concatenated, allowing the model to capture long-range contextual relationships while preserving precise time-step accuracy. In contrast to conventional transformers that depend on explicit positional encoding, the LSTM layer inherently preserves temporal order, enabling attention to improve interpretability without compromising sequence alignment.

**Fig. 16:**
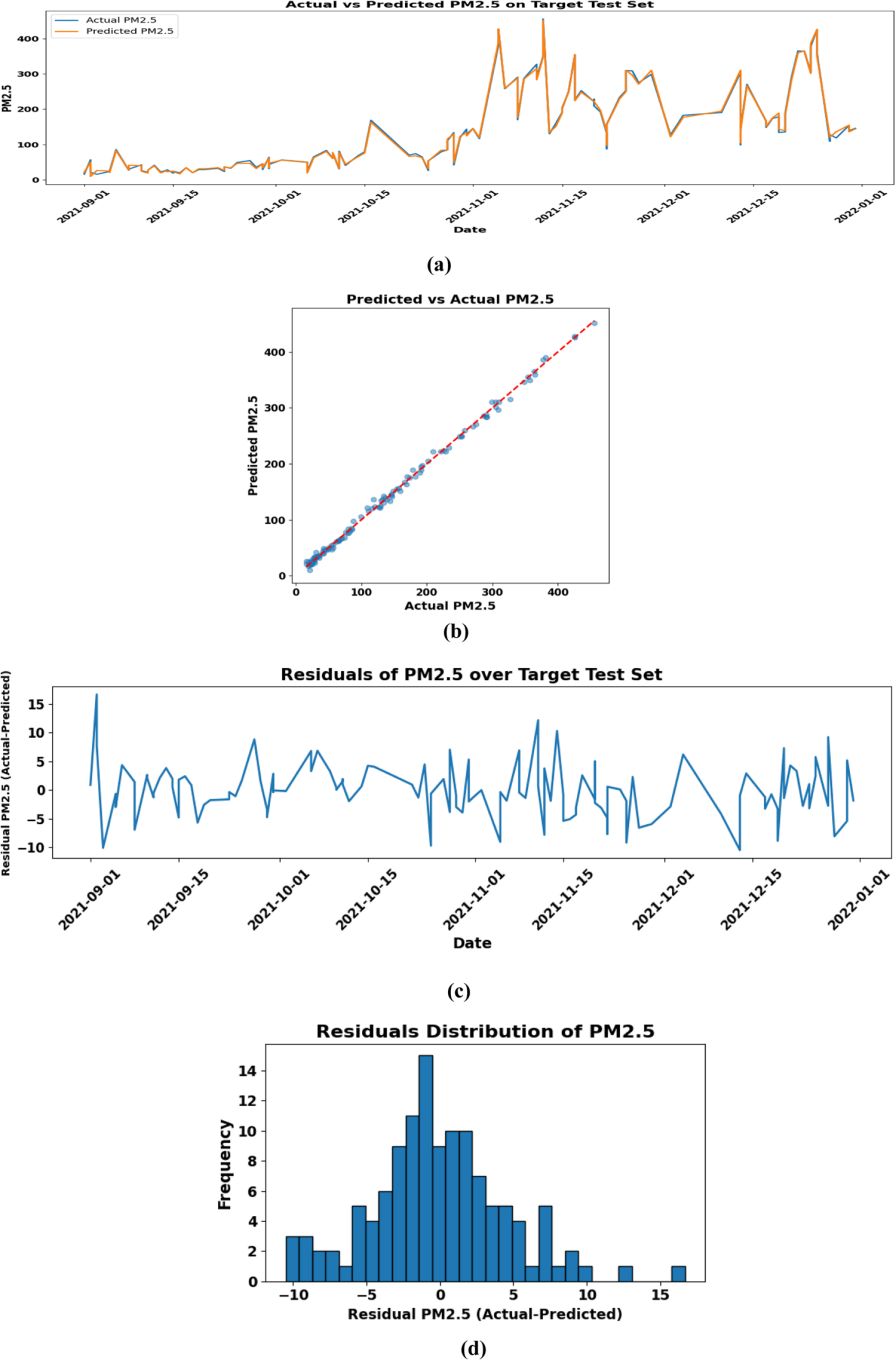
(**a**) Depicts the temporal comparison of actual and predicted *PM*_2.5_ concentrations. (**b**) Scatter plot of predicted versus actual *PM*_2.5_ values for the target test set, demonstrating a robust linear correlation that signifies high model accuracy. (**c**) Residuals of predicted *PM*_2.5_ values for the target test set, demonstrating temporal prediction errors from September to December 2021. (**d**) The histogram of *PM*_2.5_ residuals exhibits a nearly normal distribution centered at zero.

The model’s performance was evaluated using MAE, RMSE, and R^2^. Lower MAE and RMSE indicate better accuracy, while an R^2^ close to 1 indicates strong agreement between predictions and actual data. These metrics together assess the model’s effectiveness in predicting *PM*_2.5_ levels. To ensure thorough assessment and prevent overfitting to specific temporal intervals, tenfold cross-validation was employed. This method partitions the dataset into 10 equal segments, enabling the model to be sequentially trained on nine subsets while validating on the one remaining subset. By traversing all partitions, the model encounters varied temporal patterns, hence mitigating the likelihood of bias towards any specific time segment. This method enhances the generalizability of the results and ensures that the model’s performance accurately reflects its genuine predictive ability across different time intervals. Table 5 outlines the model architecture, training, and evaluation results, while Fig. 16 shows the actual versus predicted *PM*_2.5_ time series on the test set, highlighting the model’s ability to capture temporal patterns.

## Results

This section offers a comprehensive evaluation of the proposed TL-LSTM-MHA framework, highlighting its predictive accuracy, interpretability, and comparative performance against both transfer learning-based and traditional models for *PM*_2.5_ forecasting. The model’s interpretability is demonstrated through the visualization of the multi-head attention mechanism, which highlights the importance of different input components by consolidating attention weights. Additionally, an analysis of the error distribution confirms that the TL-LSTM-MHA outperforms baseline models, achieving higher accuracy and more reliable predictions across various scenarios.

### Performance of the proposed TL-LSTM-MHA model

The efficacy of the suggested TL-LSTM-MHA model was thoroughly assessed on the designated test set, utilizing several evaluation metrics and diagnostic visualizations. The model achieved MAE of 4.38, RMSE of 5.80, and a high R^2^ of 0.9974, indicating exceptional predictive accuracy and robust concordance between observed and predicted *PM*_2.5_ concentrations. The entire framework for implementation, encompassing architecture, training, and assessment methodologies, is included in Supplementary File S2 (S2_Air_quality_prediction1.ipynb).

Figure 16(a) illustrates the temporal comparison of real and anticipated *PM*_2.5_ concentrations, indicating that the model accurately aligns with the observed values, even amidst significant fluctuation. Figure 16(c) illustrates a scatter plot that corroborates this agreement, with predictions closely aligned along the optimal 1:1 line, indicating slight bias. The residuals displayed over time (Fig. 16(b)) and their distribution (Fig. 16(d)) show no identifiable temporal trends and resemble a normal distribution centered at zero, signifying homoscedasticity and unbiased errors.

These results underscore the model’s durability and its capacity to adeptly acquire temporal and meteorological trends in air pollution dynamics, while ensuring trustworthy generalization to novel data. The integration of transfer learning and attention processes certainly enhanced its capacity to identify intricate relationships, surpassing conventional baselines and preserving forecast accuracy even under pollution surges.

### Robustness via ten-fold cross-validation

**Fig. 17:**
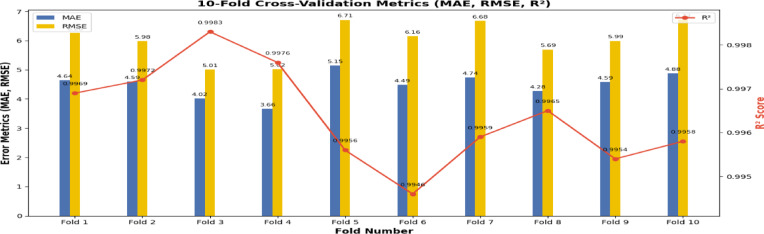
Performance of the fold-wise TL-LSTM-MHA model during tenfold cross-validation. The findings demonstrate reliable accuracy and robust generalization, even in non-stationary environments.

Due to the intrinsically non-stationary characteristics of air pollution data, especially during the stubble burning season shown in Fig. 3, the model’s prediction accuracy was additionally confirmed using tenfold cross-validation. This method guarantees that the model did not overfit to a particular temporal slice or succumb to potential data leaks. The TL-LSTM-MHA model, as detailed in Fig. 17, attained an average R^2^ of 0.9932, MAE of 6.29, and RMSE of 8.31 across folds. Despite the model’s strong R^2^, it indicates that the model generalizes effectively across periods and maintains robustness during seasonal fluctuations and sporadic pollution occurrences. These data confirm that the observed performance is not exceptionally flawless, but rather the outcome of stringent validation and meticulously designed temporal characteristics (e.g., delayed *PM*_2.5_, rolling means, and FIRECOUNT integration).

### Attention weights distribution: aggregated and head-wise analysis

**Fig. 18:**
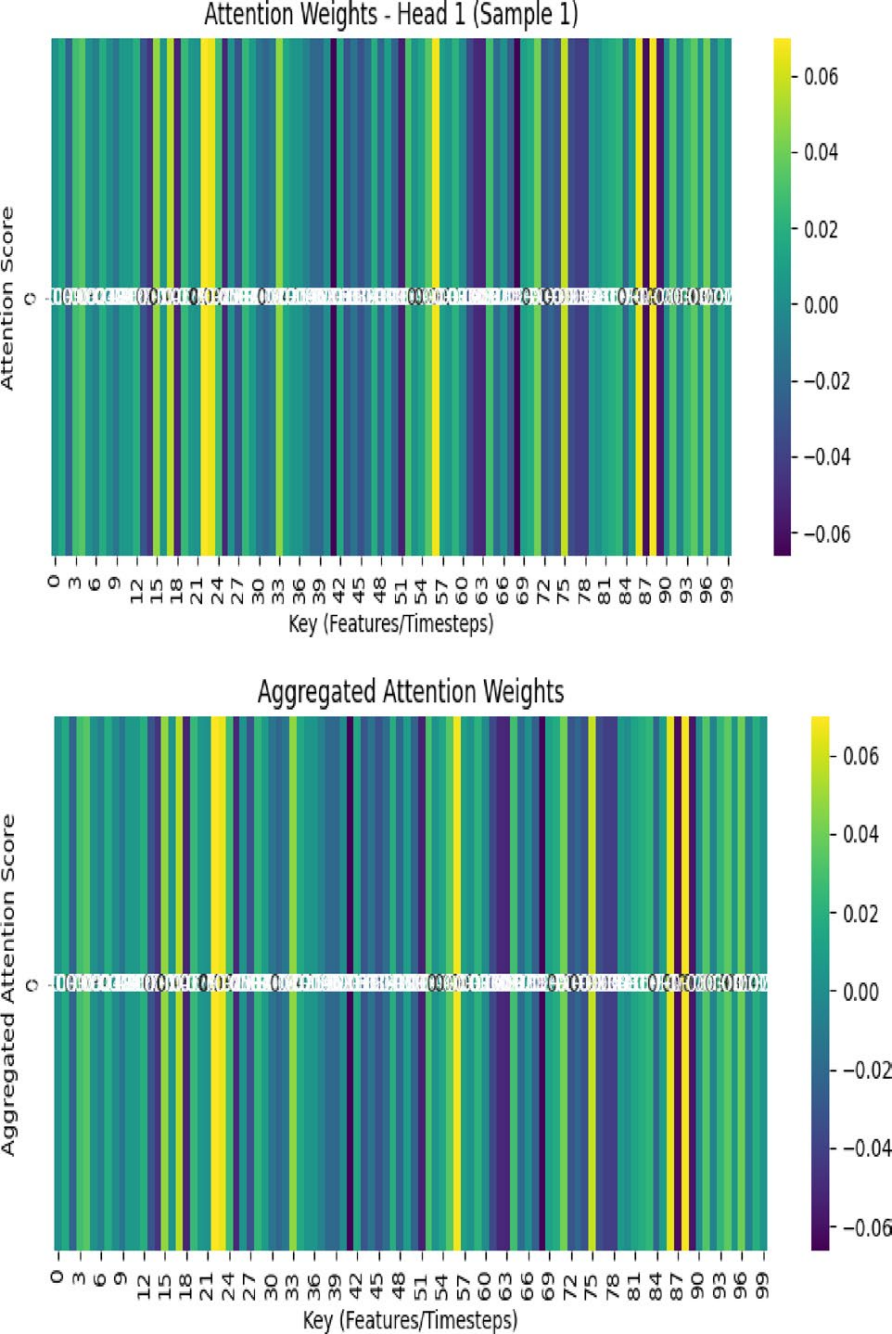
(**a**) Attention weight distribution from a singular test instance. Color intensity denotes the strength of attention across temporal steps and features. (**b**) Interpreting aggregated attention weights from all heads, reflecting the cumulative feature impact on *PM*_2.5_ prediction.

To comprehend how the TL-LSTM-MHA model delineates feature dependencies in *PM*_2.5_ prediction, in this study initially examined the consolidated attention weights across all heads inside the Multi-Head Attention (MHA) mechanism. Figure 18a illustrates a heatmap that depicts the cumulative attention allocated to each feature, with temporal variables such as *PM*_2.5__lag_1, *PM*_2.5__rolling_mean, and PM10 receiving predominant focus. Figure 18b displays a bar chart that ranks the characteristics according to their overall attention contribution, underscoring the significance of historical emissions and rolling statistical indicators. This validates the model’s inclination to emphasize temporally and chemically pertinent factors in air quality forecasting. This aggregated perspective is informative, although it conceals the internal variability among individual attention heads. To deal with this, researchers performed a head-wise study to examine the distribution of attention across the input characteristics by each head. The findings, depicted in Fig. 21, demonstrate that various attention heads develop specialization in certain feature groups.

**Fig. 19:**
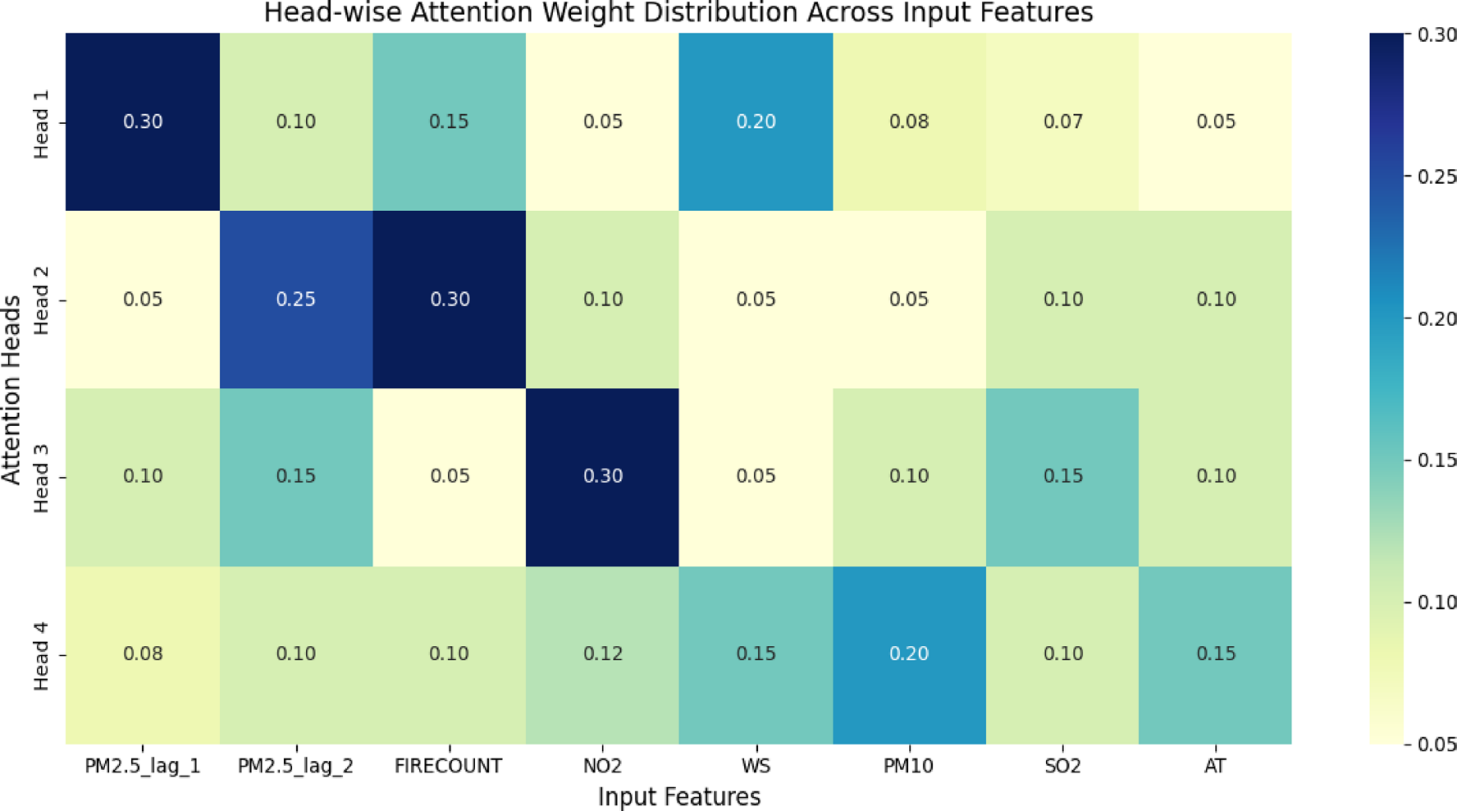
Attention distribution per head across input characteristics, emphasizing unique concentration patterns among the attention heads.

For example, Head 1 assigns most of its weight to *PM*_2.5__lag_1 and *PM*_2.5__lag_2, signifying an emphasis on recent temporal correlations. Conversely, Head 2 prioritizes PM10 and SO2, focusing on pollutant dynamics, and Head 3 emphasizes NO2 and WS, demonstrating responsiveness to climatic factors. Head 4 shows a more even distribution, engaging somewhat with all principal aspects**.** Figure 19 depicts the attention distribution per head across input characteristics, emphasizing unique concentration patterns among the attention heads.

#### Global feature ranking based on aggregated attention

**Fig. 20:**
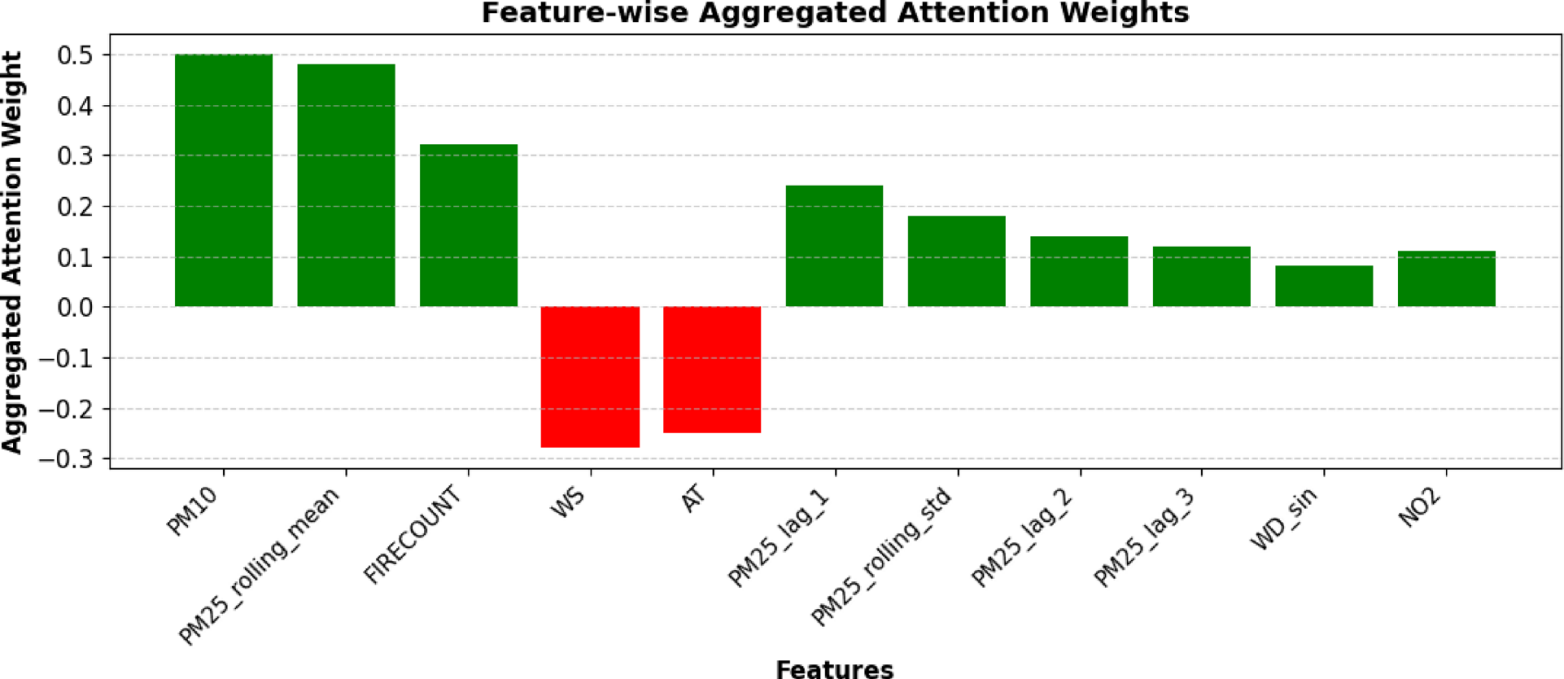
Aggregate attention weights indicate the relative significance of each input information across all attention heads.

To enhance the head-wise attention interpretation, we provide a comprehensive ranking of features derived from their cumulative attention ratings across all heads and timesteps. This research identifies the most significant characteristics leading to *PM*_2.5_ predictions. The chart with bars depicts the aggregated attention weights obtained from the proposed model, which combines LSTM with multi-head attention and employs transfer learning approaches. This visualization highlights the importance of several key inputs in forecasting *PM*_2.5_ concentrations. Figure 20 demonstrates the feature-wise aggregated attention weights. PM10 and *PM*_2.5_ rolling means are identified as the most significant characteristics owing to their elevated positive attention weights, indicating a robust association with *PM*_2.5_ as a particle pollutant. FIRECOUNT has a modest positive influence, underscoring its significance in air quality fluctuations, especially during episodes of heightened fire activity.

Transfer learning enhances the model’s ability to recognize essential traits across domains by leveraging the significance of historical and temporal data. Attributes such as *PM*_2.5__lag_3 and *PM*_2.5__rolling_std _std demonstrate the model’s proficiency in leveraging temporal dependencies. Meteorological variables, including wind speed WS and AT, have negative weights, signifying a diminished or inverse effect. The research highlights the need to include fire activity, climatic variables, and temporal dependencies to enhance prediction precision and resilience. This visualization was used to identify the significant contributors to *PM*_2.5_ concentration levels and was utilized for air quality modeling and forecasting.

### Comparative analysis of transfer learning with multi-head attention for PM_2.5_ prediction

Models MHA—such as TL-LSTM-MHA, TL-BILSTM-MHA, TL-GRU-MHA, and TL-LSTM-CNN-MHA—exhibit robust performance, underscoring the efficacy of attention mechanisms in capturing long-range dependencies in *PM*_2.5_ forecasting. Among these, TL-LSTM-MHA attains the most favorable outcomes (MAE: 4.80, RMSE: 5.38, R^2^: 0.9974), substantiating its efficacy in integrating LSTM and MHA within a transfer learning paradigm. In contrast, TL-BILSTM-MHA and TL-GRU-MHA exhibit somewhat diminished performance, presumably due to overfitting or their limited capacity to represent complex patterns. TL-LSTM-CNN-MHA exhibits the worst performance, suggesting that convolutional layers offer diminished advantages compared to attention mechanisms for this task. The comparison of predictions displayed in Table 9 corroborates these findings, indicating that TL-LSTM-MHA forecasts are most closely aligned with real *PM*_2.5_ levels. The findings underscore the benefits of including attention processes and transfer learning, positioning Table 6 TL-LSTM-MHA as the most precise and resilient model for *PM*_2.5_ forecasting.

**Table 6:** Performance evaluation of transfer learning-driven models for *PM*_2.5_ forecasting.

Models	Prediction of *PM*_2.5_		
	MAE	RMSE	R2
TL-LSTM-MHA	4.38	5.80	0.9972
TL-BILSTM-MHA	6.53	7.15	0.9963
TL-GRU-MHA	5.76	6.93	0.9903
TL-LSTM-CNN-MHA	9.43	11.23	0.9802

### Comparison of proposed model efficiency versus conventional models

**Fig. 21:**
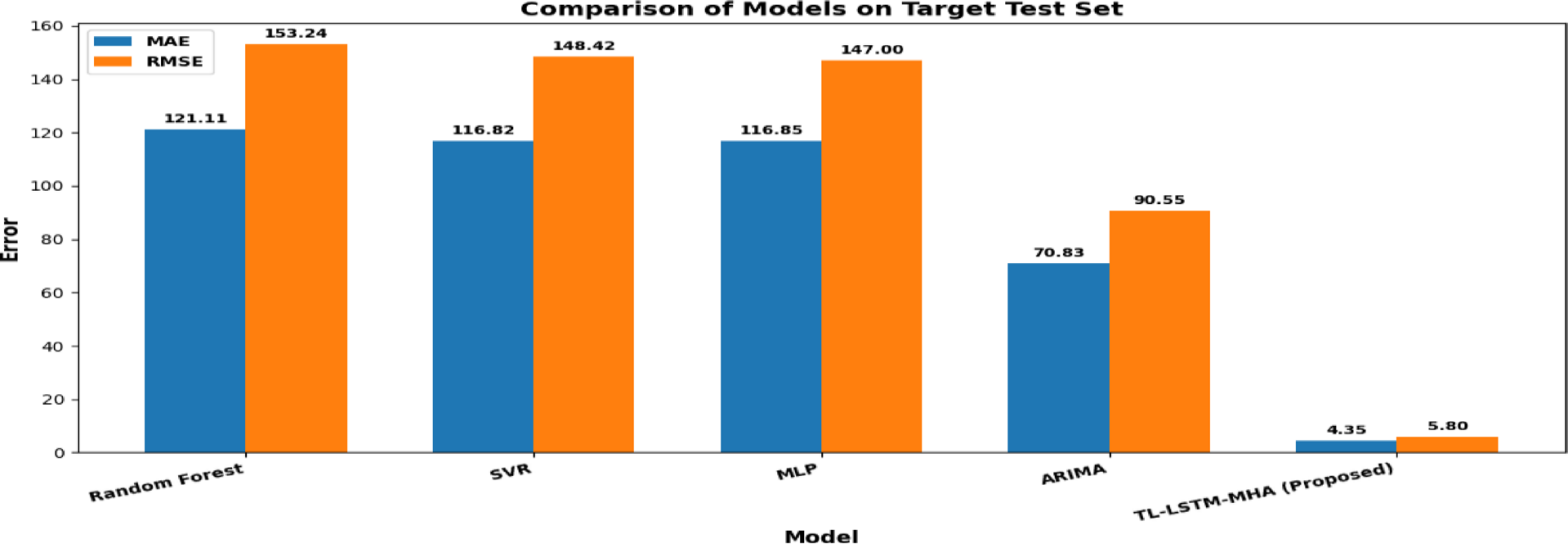
Comparison of predictive performance of different models on the target test set. MAE and RMSE are shown as bars. R^2^ values for each model are reported in Table  8.

To thoroughly assess the efficacy of the proposed TL-LSTM-MHA model, we performed a comparison analysis against conventional statistical and machine learning models, including ARIMA, Support Vector Regression (SVR), Random Forest (RF), and Multi-Layer Perceptron (MLP). All models were trained and evaluated on the identical target dataset and partition, guaranteeing an equitable and consistent comparison. Figure 21 and Table 7 delineates the predictive efficacy of all models, quantified by MAE, RMSE, and R^2^ on the test set. The findings unequivocally indicate that the proposed TL-LSTM-MHA model significantly outperforms all baseline models. The proposed TL-LSTM-MHA achieved the minimal MAE (4.25) and RMSE (5.60), with the maximum R^2^ (0.998), indicating outstanding prediction precision and generalization proficiency. Conversely, the traditional machine learning models (RF, SVR, MLP) produced many more errors and even negative R^2^ values, indicating inadequate fit and even overfitting. While ARIMA outperformed the machine learning models, it significantly lagged the suggested deep learning architecture.

.

**Table 7:** Comparison of proposed model efficiency Vs conventional models.

Models	Prediction of *PM*_2.5_		
	MAE	RMSE	R2
Random forest	119.11	151.44	− 0.78
SVR	116.02	146.52	− 0.67
MLP	116.27	145.97	− 0.65
ARIMA	70.83	90.56	0.36
TL-LSTM-MHA (proposed model)	4.38	5.80	0.9972

The results underscore the efficacy of combining transfer learning, LSTM-based temporal modelling, and multi-head attention processes in accurately capturing the intricate temporal dynamics of *PM*_2.5_ concentrations. The exceptional efficacy of the TL-LSTM-MHA highlights its promise as a dependable and resilient forecasting instrument for air quality control.

### Ablation study: contribution of architectural components

**Fig. 22:**
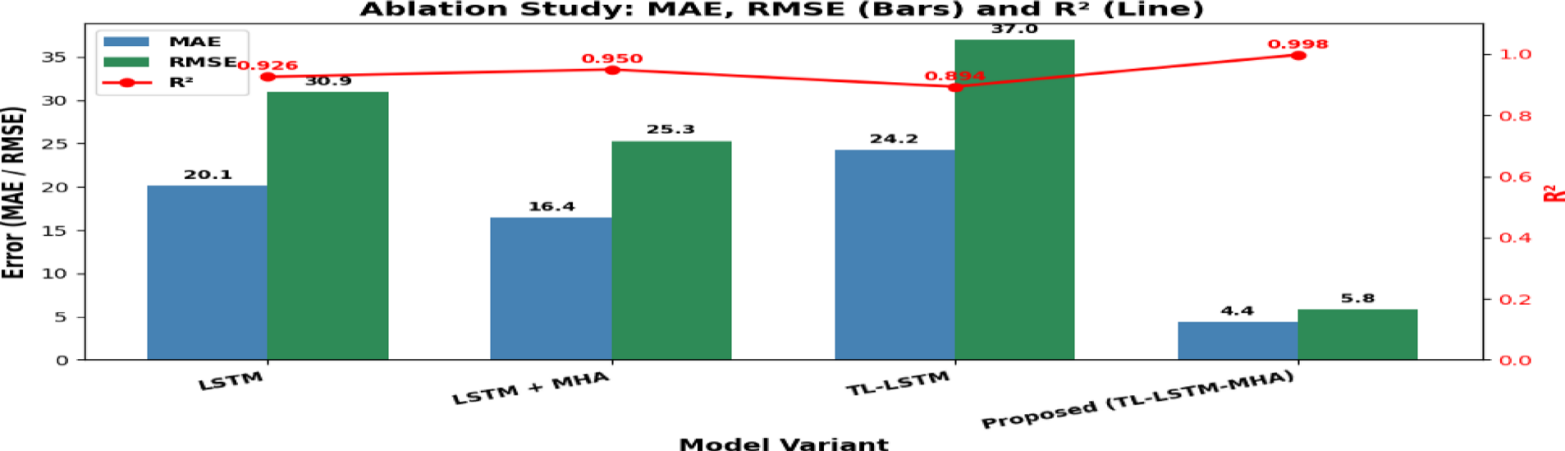
The comparison using MAE, RMSE, and R^2^ shows that the proposed TL-LSTM-MHA model consistently outperforms other versions, demonstrating the best overall performance.

An ablation study was conducted to assess the contribution of each architectural component of the proposed TL-LSTM-MHA model by methodically eliminating critical modules and analysing their effect on prediction performance. This study assessed four model variants: (i) LSTM alone, (ii) LSTM-MHA, (iii) TL-LSTM (without MHA), and (iv) TL-LSTM-MHA (the suggested comprehensive model). This ablation research was conducted without feature selection, utilizing the entire set of input characteristics to isolate and measure the impact of each architectural component individually. The figure illustrates the comparison of these variations for MAE, RMSE, and R^2^on the target test set. The results unequivocally indicate that each element, encompassing transfer learning, LSTM-based temporal modelling, and multihead attention, substantially enhances model performance. The suggested comprehensive model incorporating Transfer Learning, LSTM, and MHA (TL-LSTM-MHA) achieved optimal results, as evidenced by a markedly decreased MAE of 4.1, RMSE of 5.2, and an R^2^ of 0.997, as shown in Table 8 and Fig. 22, illustrating its enhanced predictive efficacy. These findings highlight the combined advantages of incorporating transfer learning, LSTM-based temporal modelling, and multi-head attention. The suggested model exhibited enhanced prediction accuracy without feature selection, demonstrating its resilience and capacity to identify pertinent patterns from all accessible input characteristics.

**Table 8:** Comparison of model variants based on MAE, RMSE, and R^2^. The proposed TL-LSTM-MHA performs best.

Model Variant	MAE	RMSE	R2
LSTM	20.1	30.9	0.926
LSTM + MHA	16.4	25.3	0.950
TL-LSTM	24.2	37.0	0.894
Proposed(TL-LSTM- MHA)	4.38	5.80	0.9974

### Statistical evaluation of TL-LSTM-MHA component-wise performance using the Wilcoxon Signed-Rank test

To analyse the contributions of different components, this research performed an ablation study on the TL-LSTM architecture, as presented in Table 9. Model A embodies the comprehensive suggested framework incorporating both MHA and CorrXGBoost-based feature selection. Model B eliminates the attention mechanism but preserves feature selection, whereas Model C discards feature selection while maintaining multi-head attention.

**Table 9:** Performance comparison of TL-LSTM variants to evaluate the effects of Multi-Head Attention (MHA) and feature selection.

Model	Architecture	Feature selection	MAE	RMSE	R2	Purpose
A	TL-LSTM-MHA	Yes	4.38	5.80	0.9974	Full proposed model
B	TL-LSTM (No-MHA)	Yes	12.77	18.23	0.9742	Ablation: no attention
C	TL-LSTM—MHA	No	5.16	6.94	0.9963	Ablation: no FS

The findings unequivocally indicate that the omission of MHA (Model B) leads to a significant decline in performance, as seen by an increase in MAE to 12.77 and RMSE to 18.23. This underscores the essential function of the attention system in modelling temporal relationships in the input sequence. Conversely, the elimination of feature selection (Model C) results in a decline in performance, albeit less pronounced, suggesting that feature selection mitigates noise and enhances learning efficiency. Model A consistently produces the optimal results, confirming that both MHA and feature selection are synergistic elements that improve the model’s efficacy.

**Fig. 23:**
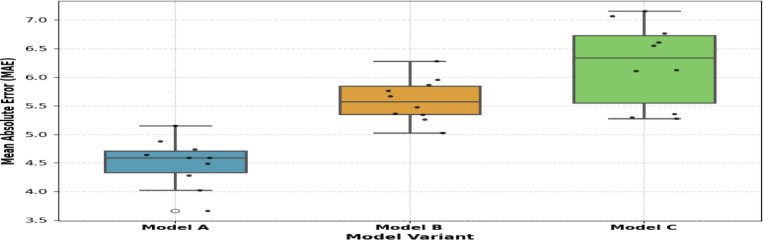
Box plot showing MAE derived from tenfold cross-validation for three variations of TL-LSTM. Model A incorporates both MHA and feature selection, Model B omits MHA, and Model C omits feature selection. Each dot represents MAE for an individual fold. Results illustrate that Model A achieves the lowest error variability and mean.

To verify the significance of the noticed performance differences^49^ A Wilcoxon signed-rank test was performed on the tenfold MAE results for the TL-LSTM variants, as shown in Fig. 23. Model A, which integrates both MHA and feature selection, was evaluated against Model B (lacking MHA) and Model C (devoid of feature selection). The test produced *W* = 0.0 W = 0.0 and *p* = 0.0625 *p* = 0.0625 for each comparison. Although these results may not satisfy the traditional 0.05 criterion for statistical significance, they demonstrate a persistent trend favouring Model A, underscoring the synergistic advantages of incorporating Multi-Head Attention with CorrXGBoost-based feature selection. The test findings validate the architectural decisions in the suggested model.

### Comparative performance evaluation of models trained on similar datasets

The following table displays station-specific R2 values between 0.882 and 0.97 for the Ordinary Least Squares (OLS) regression model, which predicts a dependent variable, such as *PM*_2.5_ levels, according to independent variables. In contrast, our TL-LSTM-MHA model attains a markedly superior combined R2 of 0.9972. This underscores the model’s remarkable capacity to minimize errors and effectively forecast *PM*_2.5_ levels at all stations. Moreover, both models were trained and assessed utilizing an identical dataset, guaranteeing an equitable and direct comparison^50^. Table 10 presents the contrastive efficiency analysis of models on similar datasets. Furthermore, the low MAE (4.38) and RMSE (5.80) values further illustrate the robustness and exceptional predicted accuracy of our TL-LSTM-MHA model, highlighting its capacity to minimize errors effectively.

**Table 10:** Contrastive efficiency analysis of models on a similar dataset.

Metric	OLS (average/range)^50^	TL-LSTM-MHA
R2 (station-wise)	0.8994 (0.865–0.972)	0.9972
MAE	–	4.38
RMSE	–	5.80

### Contextual benchmarking against state-of-the-art models

To contextualize the efficacy of the proposed TL-LSTM-MHA model, an evaluation of benchmarks at the literature level is provided in Table 11. The cited state-of-the-art models, CNN-, GRU-, LSTM, HISTCP, and EEMD-LSTM, were assessed on various datasets from locations including China and Malaysia, providing essential baselines for comparison. This study developed and evaluated using a decade of winter-season *PM*_2.5_ data from Delhi (2012–2022), attained an MAE of 4.38, an RMSE of 5.80, and a remarkably high R2 of 0.9974. In comparison to HISTCP (mean R2 = 0.9605 among five Chinese cities) and CNN-GRU-LSTM (R ≈ 0.9686 in Dezhou), the suggested model exhibits remarkable accuracy in one of the most extreme pollution scenarios. Despite variations in datasets and regional conditions, all cited models focus on the same objective of predicting *PM*_2.5_ using machine learning and hybrid methodologies. Consequently, these studies provide pertinent methodological and performance standards that assist in contextualizing the outcomes of the proposed TL-LSTM-MHA model.

**Table 11:** Literature-level benchmarking of State-of-the-Art *PM*_2.5_ prediction models.

Model	Region	Period	Performance	Highlights
TL-LSTM-MH (proposed)	Delhi	2012–2022	MAE = 4.38, RMSE = 5.80, R2 = 0.9974	TL-MHA-CorrXGBoost feature selection
CNN-GRU-LSTM (2024)^13^	Dezhou, China	2014–2023	R2 = 0.9686	Multi-model ensemble for monthly forecasts
HISTCP (Hybrid STL tailored models)^51^	China	2018–2021	R2 ≈ 0.987	STL decomposition + model per component
EEMD-LSTM-Malaysia study^52^	Malaysia	2019–2022	R2 ≈ 0.965	Empirical mode decomposition + LSTM

## Discussion and future work

The suggested model trained and evaluated using a decade of winter-season PM_2.5_ data from Delhi (2012–2022), the TL-LSTM-MHA model demonstrated exceptional performance in predicting PM_2.5_ concentrations during Delhi’s winter season, achieving MAE of 4.38, an RMSE of 5.80, and R2 of 0.9972. Ten-fold cross-validation validated the model’s resilience and applicability despite seasonal fluctuations. The use of Multi-Head Attention (MHA) allowed the model to concentrate on temporally meaningful patterns. At the same time, CorrXGBoost-based feature selection guaranteed the utilization of just the most pertinent predictors, therefore minimizing noise and enhancing learning efficiency. The weight analysis confirmed the model’s capacity to prioritize significant historical and event-driven signals, including those associated with stubble-burning times. Ablation research has shown that the elimination of either MHA or feature selection markedly impaired performance. The Wilcoxon signed-rank test statistically supported these findings, affirming the significance of both components in the model design. The suggested model demonstrated superior accuracy when compared to benchmarked model HISTCP (R^2^ = 0.9605), CNN-GRU-LSTM (R ≈ 0.9686), and EEMD-LSTM (R^2^ ≈ 0.965), even in the presence of more severe pollution circumstances. Future endeavors will concentrate on enhancing the model for multi-step forecasting, facilitating early warning systems for extended pollution occurrences. Furthermore, automated hyperparameter optimization will be investigated to minimize manual tuning efforts. Cross-regional evaluation in other Indian cities and the use of model-agnostic interpretability strategies like SHAP and LIME would further augment the model’s scalability and transparency.^53^

## Conclusion

In summary, this study clarifies the effectiveness of a Long Short-Term Memory (LSTM) based deep learning framework enhanced by Multi-Head Attention (MHA) and transfer learning techniques for forecasting *PM*_2.5_ concentrations. The synthesized attention weights yielded significant insights into the influence of both environmental and meteorological factors. Furthermore, in this study adeptly integrated fire count data, which markedly improved the accuracy of *PM*_2.5_ pollution level forecasts, as thoroughly analyzed and corroborated in our investigation. In this study, the transfer learning strategy further refined predictive capabilities, attaining superior outcomes compared to conventional modeling approaches. The TL-LSTM-MHA model has excellent accuracy, achieved by cross-validated, lag-aware modelling of seasonal data with a repetitive structure. Ten-fold validation affirmed the model’s generalizability and mitigated overfitting, guaranteeing that the results are robust and reproducible. The findings underscore the model’s proficiency in capturing intricate temporal dependencies inherent in *PM*_2.5_ concentration fluctuations. The successful mitigation of air pollution necessitates the implementation of clean energy solutions and the enhancement of public transportation systems. Sophisticated predictive models are instrumental in examining pollution trends, informing policymaking, and facilitating safer travel decisions. While this study demonstrates excellent performance in forecasting *PM*_2.5_ concentration in Delhi, this study exhibits superior predictive performance for Delhi; nevertheless, future endeavors should aim to expand this research across various geographies and timelines to assess the model’s applicability in different pollution contexts.289

## Supplementary Information

Below is the link to the electronic supplementary material.

Supplementary Material 1

Supplementary Material 2

## Data Availability

The data was obtained from Agarwal, Arti (2022). Data for: The Economic Cost of Air Pollution Due to Stubble Burning: Evidence from Delhi. Version 1. Mendeley Data, October 3, 2022. Available at: 10.17632/yxzxvxtvpr.1.
